# The causal association between smoking initiation, alcohol and coffee consumption, and women’s reproductive health: A two-sample Mendelian randomization analysis

**DOI:** 10.3389/fgene.2023.1098616

**Published:** 2023-04-06

**Authors:** Zhaoying Jiang, Renke He, Haiyan Wu, Jiaen Yu, Kejing Zhu, Qinyu Luo, Xueying Liu, Jiexue Pan, Hefeng Huang

**Affiliations:** ^1^ International Institutes of Medicine, The Fourth Affiliated Hospital, Zhejiang University School of Medicine, Yiwu, China; ^2^ Key Laboratory of Reproductive Genetics (Ministry of Education), Department of Reproductive Endocrinology, Women’s Hospital, Zhejiang University School of Medicine, Hangzhou, China; ^3^ Obstetrics and Gynecology Hospital, Institute of Reproduction and Development, Fudan University, Shanghai, China; ^4^ Research Units of Embryo Original Diseases, Chinese Academy of Medical Sciences, Shanghai, China; ^5^ Shanghai Key Laboratory of Embryo Original Diseases, Shanghai, China

**Keywords:** Mendelian randomization, smoking initiation, alcohol drinking, coffee consumption, reproduction-related hormones, menstrual health

## Abstract

**Objective:** A number of epidemiological studies have demonstrated that smoking initiation and alcohol and coffee consumption were closely related to women’s reproductive health. However, there was still insufficient evidence supporting their direct causality effect.

**Methods:** We utilized two-sample Mendelian randomization (TSMR) analysis with summary datasets from genome-wide association study (GWAS) to investigate the causal relationship between smoking initiation, alcohol and coffee consumption, and women’s reproductive health-related traits. Exposure genetic instruments were used as variants significantly related to traits. The inverse-variance weighted (IVW) method was used as the main analysis approach, and we also performed MR-PRESSO, MR-Egger, weighted median, and weighted mode to supplement the sensitivity test. Then, the horizontal pleiotropy was detected by using MRE intercept and MR-PRESSO methods, and the heterogeneity was assessed using Cochran’s Q statistics.

**Results:** We found evidence that smoking women showed a significant inverse causal association with the sex hormone-binding globulin (SHBG) levels (corrected *β* = −0.033, *p* = 9.05E-06) and age at menopause (corrected *β* = −0.477, *p* = 6.60E-09) and a potential positive correlation with the total testosterone (TT) levels (corrected *β* = 0.033, *p* = 1.01E-02). In addition, there was suggestive evidence for the alcohol drinking effect on the elevated TT levels (corrected *β* = 0.117, *p* = 5.93E-03) and earlier age at menopause (corrected *β* = −0.502, *p* = 4.14E-02) among women, while coffee consumption might decrease the female SHBG levels (corrected *β* = −0.034, *p* = 1.33E-03).

**Conclusion:** Our findings suggested that smoking in women significantly decreased their SHBG concentration, promoted earlier menopause, and possibly reduced the TT levels. Alcohol drinking had a potential effect on female higher TT levels and earlier menopause, while coffee consumption might lead to lower female SHBG levels.

## 1 Introduction

Tobacco, alcohol, and coffee are common lifestyles, and their consumption has increased during the COVID-19 lockdown (Vanderbruggen et al., 2020; González-Monroy et al., 2021; Bakaloudi et al., 2022). However, the consumption growth of the three lifestyles are associated with an increased risk of developing major health problems including cardiovascular disease (Bhatnagar, 2017) and immune function-related diseases (Rychter et al., 2021). Furthermore, in particular, studies have suggested that smoking is associated with adverse reproductive events in women, such as reproductive function, folliculogenesis, steroidogenesis, and embryo transport (Dechanet et al., 2011; Sharma et al., 2013; Wikoff et al., 2017; Oostingh et al., 2019). Alcohol drinking and over-consumption of coffee have also been studied in relation to female reproductive health, with some studies suggesting a potential negative impact on fertility and pregnancy outcomes (Sharma et al., 2013; Wikoff et al., 2017; Oostingh et al., 2019). Furthermore, disturbances in sex hormones and menstrual health in women have been reported as the manifestations of reproductive diseases, with nearly 26.32% (500 million/1.9 billion) of women suffering from menstrual abnormality with endocrine hormone-related changes in 2018 (Babbar et al., 2022). In this study, we aimed to investigate the correlation between smoking initiation, alcohol and coffee consumption, and female reproductive health, focusing on some common and presentative sex hormones (e.g., sex hormone-binding globulin (SHBG), total testosterone (TT), bioavailable testosterone (bio-T), estradiol (E_2_), and anti-Mullerian hormone (AMH)) and menstrual phenotypes (e.g., menopausal age, irregular menstrual cycle/bleeding, and dysmenorrhea). As far as we know, currently some cross-sectional studies and reviews have reported tobacco (Wang, 2021), alcoholic beverages (Erol et al., 2019), and high caffeine (Pihan-Le Bars et al., 2017; Hang et al., 2019) consumptions were risk factors for abnormal female hormone levels, such as SHBG, TT, and E_2_. Furthermore, earlier menopausal age, irregular menstrual cycle/bleeding, and dysmenorrhea were considered to be brought about by cigarettes, alcohol drinking, and coffee consumption (Latthe et al., 2006; Hahn et al., 2013; Schoenaker et al., 2014; Onieva-Zafra et al., 2020). However, the true causal relationship remains unclear due to potential bias and residual confounding.

Mendelian randomization (MR) was a form of innovative and popular analysis that utilized single-nucleotide polymorphisms (SNPs) as genetic instruments to estimate the causal effect of an exposure on certain outcomes (Burgess et al., 2013). According to Mendel’s law, during gametogenesis and fertilization, the alleles of genetic variants were segregated randomly, which led to their independence with confounding factors such as the environment, age, and sex. This allowed us to bypass the possible and potential biases from confounding and reverse causality and strengthen the causal inference in exposure–outcome associations.

Previous MR studies have demonstrated the significant impact of smoking initiation and alcohol and coffee consumption on an individual’s health, including cardiovascular diseases (Hoek et al., 2022), Parkinson’s disease (Domenighetti et al., 2022), and female reproductive health, such as polycystic ovary syndrome (PCOS) (Larsson and Burgess, 2022), infertility (Hernáez et al., 2022), and pregnancy loss (Yuan et al., 2021). However, no robust evidence has been provided to support the correlation between the three common modern lifestyles (smoking initiation, alcohol drinking, and coffee consumption) and reproductive traits (SHBG, bio-T, TT, E_2_, and AMH) or abnormal menstrual phenotypes (age at menopause, irregular menstrual cycle/bleeding, and dysmenorrhea). Herein, we employed a two-sample MR approach to investigate the association of these three lifestyle behaviors with reproductive traits and abnormal menstrual phenotypes. We aimed to complement the existing evidence and confirm their causality by addressing the potential bias and pleiotropy effect and assess the robustness of MR findings in our study.

## 2 Materials and methods

### 2.1 Study design

Our study utilized the two-sample MR analysis with publicly available GWAS data. MR analysis requires the fulfillment of the following three hypotheses: first, the genetic instrument is supposed to be strongly associated with the exposures. Second, the genetic instrument selected is independent of confounders. Third, the genetic instrument should not affect the outcome except *via* the way of the exposure (Bowden et al., 2015). Additionally, to fulfill the requirements of the two-sample MR analysis requirements, the summary statistics for both exposures and outcomes in our study were collected from different sources and consortia.

### 2.2 Genetic instrument selection

First, to collect genetic instruments for our analysis, we extracted 378 and 99 single-nucleotide polymorphisms (SNPs) associated with smoking initiation (whether smoking regularly) and alcohol drinking (alcohol drinking per week), respectively, from a recent largest and available meta-analysis of 33 GWAS, involving up to 1.2 million individuals (Liu et al., 2019). Likewise, 10 SNPs associated with coffee consumption (coffee consumption per day) were sourced from a trans-ethnic meta-analysis of GWAS from 28 studies, involving up to 129,488 individuals (Cornelis et al., 2015), with only European ancestry data selected as genetic instruments after excluding African–American studies. Second, we adjusted for age, sex, and major genetic principal components in our association tests and selected SNPs at the genome-wide significance level (*p* < 5.0 × 10^−8^) to ensure that SNPs were strongly associated with their exposure phenotypes. Then, we ensured independence of all instrumental variants (IVs) by performing a clumping procedure (R2 = 0.01, kb = 10,000) and removing the linkage disequilibrium between SNPs, based on the European data from the 1000 Genomes Project (Smith and Ebrahim, 2003; Auton et al., 2015). Finally, to avoid weak instrument bias, we calculated the F-statistics of each genetic instrument (SNP) (Li and Martin, 2002; Palmer et al., 2012) and ensured strong effect sizes by selecting instruments with F-statistic values larger than 10 in our MR analysis. In conclusion, we finally selected 314 SNPs as smoking initiation IVs, 84 SNPs as alcohol drinking IVs, and 4 SNPs as coffee consumption IVs, and these instruments have been used in previous MR studies (Georgiou et al., 2021; Yuan et al., 2021; Zhou et al., 2022). In addition, overview information of our selected instruments is listed in Table 1, with additional details provided in Supplementary Table S1.

**TABLE 1:** Overview of the data sources of the instrumental variables used in the MR study.

Exposure	Unit	Participants included in analysis	Consortium	Adjustment	Identified SNP	Used instrument	PubMed ID or web link
Smoking initiation	1 SD increase in the prevalence of ever smoking	1,232,091 European-descent individuals	GWAS and Sequencing Consortium Of Alcohol And Nicotine Use (GSCAN)	Age, sex, and the first 10 principal components	378	314	30643251
Alcohol drinking	1 SD increase in log-transformed alcoholic drinks/week	941,280 European-descent individuals	GWAS and Sequencing Consortium Of Alcohol And Nicotine Use (GSCAN)	Age, sex, and the first 10 principal components	99	84	30643251
Coffee consumption	One cup increase of coffee consumed/day	129,488 individuals (6.2% African–Americans)	Coffee and Caffeine Genetics Consortium (CCGC)	Age, Sex, smoking status, case–control status, study site, family structure, and/or study-specific principal components of population substructure	10	4 (only European-descent individuals included)	25288136

Note: SNPs, single-nucleotide polymorphisms; PubMed ID, PubMed identifier; SD, standard deviation; GWAS, genome-wide association study.

### 2.3 Data sources

First, we obtained GWAS summary statistics for reproductive hormones in women from the United Kingdom Biobank (European ancestry, SHBG levels, n = 189,473; bio-T levels, n = 188,507; TT levels, n = 230,454; E_2_ levels, n = 163,985) (Ruth et al., 2019; Schmitz et al., 2021). Additionally, summary data for the AMH was used from a genome-wide meta-analysis including five cohorts with 3,344 premenopausal female European ancestry (Ruth et al., 2019). In addition to these common female-specific reproductive traits, our outcomes also focused on some abnormal menstrual phenotypes, including age at menopause, irregular menstrual cycle/bleeding, and dysmenorrhea. Summary-level genetic data for age at menopause were collected from publicly available data based on the UK Biobank cohort (Loh et al., 2018) (European ancestry, n = 156,364). The GWAS outcomes of irregular menstrual cycle/bleeding and dysmenorrhea were sourced from the UK Biobank (Jiang et al., 2021) (European ancestry, n = 247,540, with the former including 1,530 cases and 246,010 controls and the latter including 413 cases and 247,127 controls).

### 2.4 Statistical analysis

We used several MR methods to estimate the sensitivity analysis. First, the primary and main sensitivity analysis was conducted using the random-effects inverse-variance weighted (IVW) method, which was the most commonly used method in MR analysis. This method calculated the inverse-variance weighted mean of ratio estimates from two or more instruments and assumed that all SNPs used were valid. To account for potential heterogeneity in our study, we conducted random-effects IVW instead of fixed IVW, which might produce overly precise results in the presence of heterogeneity (Hartwig et al., 2017). We also used the MR pleiotropy residual sum and outlier (MR-PRESSO) as a supplementary method, which detected and corrected for horizontal pleiotropy by identifying and removing outlier genetic variants that caused bias in the IVW estimate. This embedded distortion analysis could distinguish the differences between estimates before and after removing outliers. Third, weighted median (WM), MR-Egger (MRE) regression, and weighted mode (Wm) were also used to supplement the sensitivity analysis, which ensured the stability and reliability of the results further. Among these three methods, MRE regression provided a valid estimate of the effect, even if all SNPs were invalid instruments, under the assumption that horizontal pleiotropic effects and SNP exposure associations were uncorrelated. This assumption implied that the instrument strength was independent of direct effects. In comparison to MRE regression, the WM method provided lower type I error and higher causal estimates. Furthermore, similar to the WM method, Wm estimated a causal effect by taking the mode of the effect estimates from all valid instrumental variables, but it could be more robust in situations where there were a few strong instrumental variables that had a large effect on the causal estimate (Hartwig et al., 2017). Meanwhile, the “leave-one-out” sensitivity analysis was conducted to test whether a single genetic variant was driving the causal association. Additionally, the Cochran’s Q-statistics was utilized to test heterogeneity between genetic instruments, and MR-Egger intercepts and MR-PRESSO were calculated to assess the possible horizontal pleiotropy. Finally, to help interpret the results and assess potential sources of bias, we presented the results in the scatter plots, forest plots, and funnel plots. Generally, a *p*-value < 0.05 was considered statistically significant, but we applied the Bonferroni method in our multiple-testing and adjusted the statistically significant *p*-value (*p* < 0.05/24 = 2.08E-03). If the outcomes were dichotomous variables, the estimated effects were exhibited as odds ratios (ORs), corresponding 95% confidence intervals (CIs), and *p*-values. A beta effect (β) with 95% CIs were also presented when the outcomes were continuous variables. Finally, all analyses were performed using the TwoSampleMR (Hemani et al., 2018) and MR-PRESSO (Verbanck et al., 2018) packages in R Software 4.1.2.

## 3 Results

### 3.1 Smoking and female reproductive health

**FIGURE 1:**
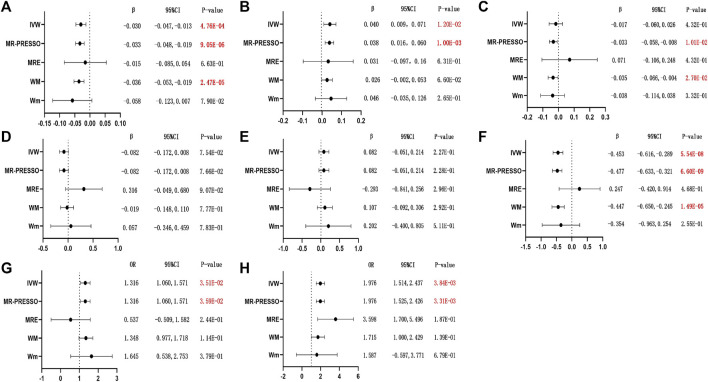
MR results of causal effects of smoking initiation SNPs on related outcome risks. (The estimates were derived from the random-effects inverse-variance weighted (IVW) model, the MR pleiotropy residual sum and outlier (MR-PRESSO) approach, MR-Egger (MRE) regression, weighted median (WM), and weighted mode (Wm). **(A–H)**: **(A)** sex hormone-binding globulin, SHBG; **(B)** bioavailable testosterone, bio-T; **(C)** total testosterone, TT; **(D)** estradiol, E_2_; **(E)** anti-Mullerian hormone, AMH; **(F)** age at menopause; **(G)** irregular menstrual cycle/bleeding; and **(H)** dysmenorrhea. Outliers of MR-PRESSO: **(A)** rs56208390, rs3115418, rs13066050, rs7631379, rs3934797, rs1116690, rs7743165, rs9331343, rs76841737, rs11192347, rs644740, rs76460663, rs1106363, rs11611651, rs62052916, rs4476253, and rs7505855; **(B)** rs56208390, rs3115418, rs13066050, rs1549979, rs1116690, rs2173019, rs7024924, rs10969352, rs11192347, rs76460663, rs62052916, and rs4476253; **(C)** rs1518393, rs3115418, rs13066050, rs1549979, rs2173019, rs359431, rs13261666, rs7024924, rs10966092, rs10969352, rs11192347, and rs76460663; **(D)** N; **(E)** N; **(F)** rs160631, rs9331343, rs10966092, and rs56902655; **(G)** N; and **(H)** N. *p*-values < 5.00E-02 are presented in red, and red and bold font means *p*-values < 2.08E-03. β, beta; CI, confidence interval; OR, odds ratio; N, none).

The smoking in women reflected lower SHBG concentrations (*β*_IVW_ = −0.030; 95% CI = −0.047, −0.013; *p* = 4.76E-04; Figure 1A) and earlier age at menopause (*β*_IVW_ = −0.453; 95% CI = −0.616, −0.289; *p* = 5.54E-08; Figure 1F). Due to the presence of horizontal pleiotropy in the causal relationship (both *p* < 0.001; Supplementary Table S2), we employed the MR-PRESSO method to identify and address the potential outliers in our study. This approach helped us identify 17 outlier SNPs (rs56208390, rs3115418, rs13066050, rs7631379, rs3934797, rs1116690, rs7743165, rs9331343, rs76841737, rs11192347, rs644740, rs76460663, rs1106363, rs11611651, rs62052916, rs4476253, and rs7505855) that could have influenced the association between smoking and SHBG concentration, as well as four outlier SNPs (rs160631, rs9331343, rs10966092, and rs56902655) that could have influenced the association between smoking and age at menopause. Outlier correction made a stronger inverse association between female smokers and the SHBG concentration and age at menopause (corrected *β*_MR-PRESSO_ = −0.033; 95% CI = −0.048, −0.019; *p* = 9.05E-06; corrected *β*_MR-PRESSO_ = −0.477; 95% CI = −0.633, −0.321; *p* = 6.60E-09; Figures 1A, F, respectively). The intercept of MR-Egger also suggested horizontal pleiotropy in age at menopause (*p* = 3.48E-02; Supplementary Table S2). In other sensitivity analyses, the weighted median approach also showed that smoking in women had a significant inverse effect on the SHBG concentration (*β*_WM_ = −0.036; 95% CI = −0.053, −0.019; *p* = 2.47E-05; Figure 1A) and age at menopause (*β*_WM_ = −0.447; 95% CI = −0.650, −0.245; *p* = 1.49E-05; Figure 1F). Although the MR-Egger and the weighted mode showed no causality of smoking with SHBG and age at menopause (Figures 1A, F, respectively).

In addition, the IVW analysis supported suggestive evidence of a positive association for female smoking and the bio-T levels, while the MR-PRESSO method could reach a corrected *p*-value of strong significance after excluding these 12 outliers (rs56208390, rs3115418, rs13066050, rs1549979, rs1116690, rs2173019, rs7024924, rs10969352, rs11192347, rs76460663, rs62052916, and rs4476253; *β*_IVW_ = 0.040; 95% CI = 0.009, 0.071; *p* = 1.20E-02 *vs*. *β*_MR-PRESSO_ = 0.038; 95% CI = 0.016, 0.060; *p* = 1.00E-03; Figure 1B). No relationship was observed between smoking and bio-T levels in the rest three alternative MR algorithms (Figure 1B). MR-Egger intercept did not show significant directional pleiotropy (Supplementary Table S2). As for the association of female smokers with TT levels, although the IVW analysis did not find any causal indication (Figure 1C), MR-PRESSO showed a potential harmful causality (corrected *β*_MR-PRESSO_ = −0.033; 95% CI = −0.058, −0.008; *p* = 1.01E-02; Figure 1C), after correcting 12 SNP outliers for the result (rs1518393, rs3115418, rs13066050, rs1549979, rs2173019, rs359431, rs13261666, rs7024924, rs10966092, rs10969352, rs11192347, and rs76460663) because of the horizontal pleiotropy (*p* < 0.001; Supplementary Table S2). In addition, the WM showed a similar result (*β*_WM_ = −0.035, 95% CI = −0.066, −0.004; *p* = 2.70E-02; Figure 1C).

The smoking initiation SNP IVW analysis showed potential causality for a positive effect on irregular menstrual cycle/bleeding (OR_IVW_ = 1.316; 95% CI = 1.060, 1.571; *p* = 3.51E-02; Figure 1G) and dysmenorrhea (OR_IVW_ = 1.976; 95% CI = 1.514, 2.437; *p* = 3.84E-03; Figure 1H). The smoking in women had a similar causality with irregular menstrual cycle/bleeding (OR_MR-PRESSO_ = 1.316; 95% CI = 1.060, 1.571; *p* = 3.59E-02; Figure 1G) and dysmenorrhea (OR_MR-PRESSO_ = 1.976; 95% CI = 1.525, 2.426; *p* = 3.31E-03; Figure 1H) after corrected by the MR-PRESSO analysis, but it did not reach the corrected *p*-value of strong significance. However, no relationship was observed between smoking and E_2_ and AMH levels (Figures 1D, E, respectively).

### 3.2 Alcohol drinking and female reproductive health

**FIGURE 2:**
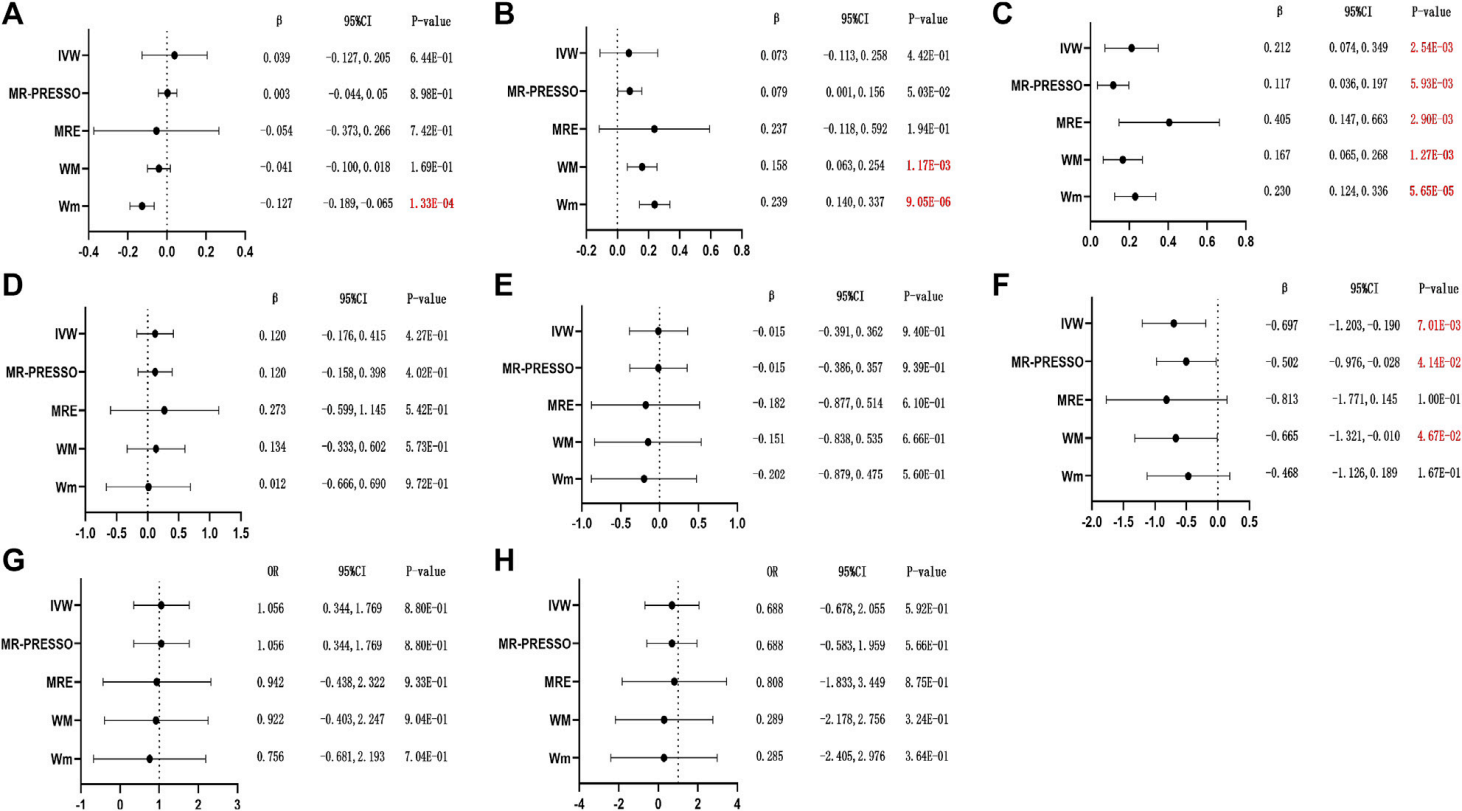
MR results of causal effects of alcohol drinking SNPs on related outcome risks. (The estimates were derived from the random-effects IVW model, the MR-PRESSO approach, MRE regression, WM, and Wm. **(A–H)**: **(A)** SHBG; **(B)** bio-T; **(C)** TT; **(D)** E_2_; **(E)** AMH; **(F)** age at menopause; **(G)** irregular menstrual cycle/bleeding; and **(H)** dysmenorrhea. Outliers of MR-PRESSO: **(A)** rs705687, rs56337305, rs13094887, rs11940694, rs6460047, rs35034355, rs1217091, rs55932213, rs7074871, rs62044525, rs7185555, and rs2532276; **(B)** rs705687, rs56337305, rs13094887, rs4501255, rs35034355, rs1217091, rs28601761, rs55932213, rs11625650, and rs62044525; **(C)** rs705687, rs13094887, rs4501255, rs35034355, rs28601761, and rs11625650; **(D)** N; **(E)** N; **(F)** rs13094887; **(G)** N; and **(H)** N. *p*-values < 5.00E-02 are presented in red, and red and bold font means *p*-values < 2.08E-03. β, beta; CI, confidence interval; OR, odds ratio; N, none).

The IVW MR results supported that a suggestive causal effect of the alcohol drinking on female elevated TT levels and earlier menopausal age (*β*_IVW_ = 0.212; 95% CI = 0.074, 0.349; *p* = 2.54E-03, and *β*_IVW_ = −0.697; 95% CI = −1.203, −0.190; *p* = 7.01E-03; Figures 2C, , respectively), whereas directional horizontal pleiotropy tests of MR-PRESSO displayed significant difference (both *p* < 0.001; Supplementary Table S2) and outlier correction also tended to exhibit a potential causal effect on the increasing TT levels and early menopause (corrected *β*_MR-PRESSO_ = 0.171; 95% CI = 0.036, 0.197; *p* = 5.93E-03 and corrected *β*_MR-PRESSO_ = −0.502; 95% CI = −0.976, −0.028; *p* = 4.14E-02; Figures 2C, F respectively) in female drinkers. Additionally, the weighted median and weighted mode approach showed a more significant association (*β*_WM_ = 0.167; 95% CI = 0.065, 0.268; *p* = 1.27E-03 and *β*_Wm_ = 0.230; 95% CI = 0.124, 0.336; *p* = 5.65E-05; Figure 2C), while the MR-Egger method yielded slightly higher effect estimates with potential association between alcohol drinking and female TT levels (*β*_MRE_ = 0.406; 95% CI = 0.147, 0.663; *p* = 2.90E-03; Figure 2C), whose intercept showed no horizontal pleiotropy (Supplementary Table S2). In the weighted median approach, female alcohol drinkers also exerted a possible negative influence on their age at menopause (*β*_WM_ = −0.665; 95% CI = −1.321, −0.010; *p* = 4.67E-02; Figure 2F), and the MRE intercept did not show significant directional pleiotropy (Supplementary Table S2). However, no causal effects were found between alcohol drinking and SHBG, bio-T, E_2_, and AMH levels (Figures 2A, B, D, E, respectively), as well as irregular menstrual cycle/bleeding and dysmenorrhea (Figures 2G, H, respectively), even after the heterogeneity and horizontal pleiotropy were eliminated.

### 3.3 Coffee consumption and female reproductive health

**FIGURE 3:**
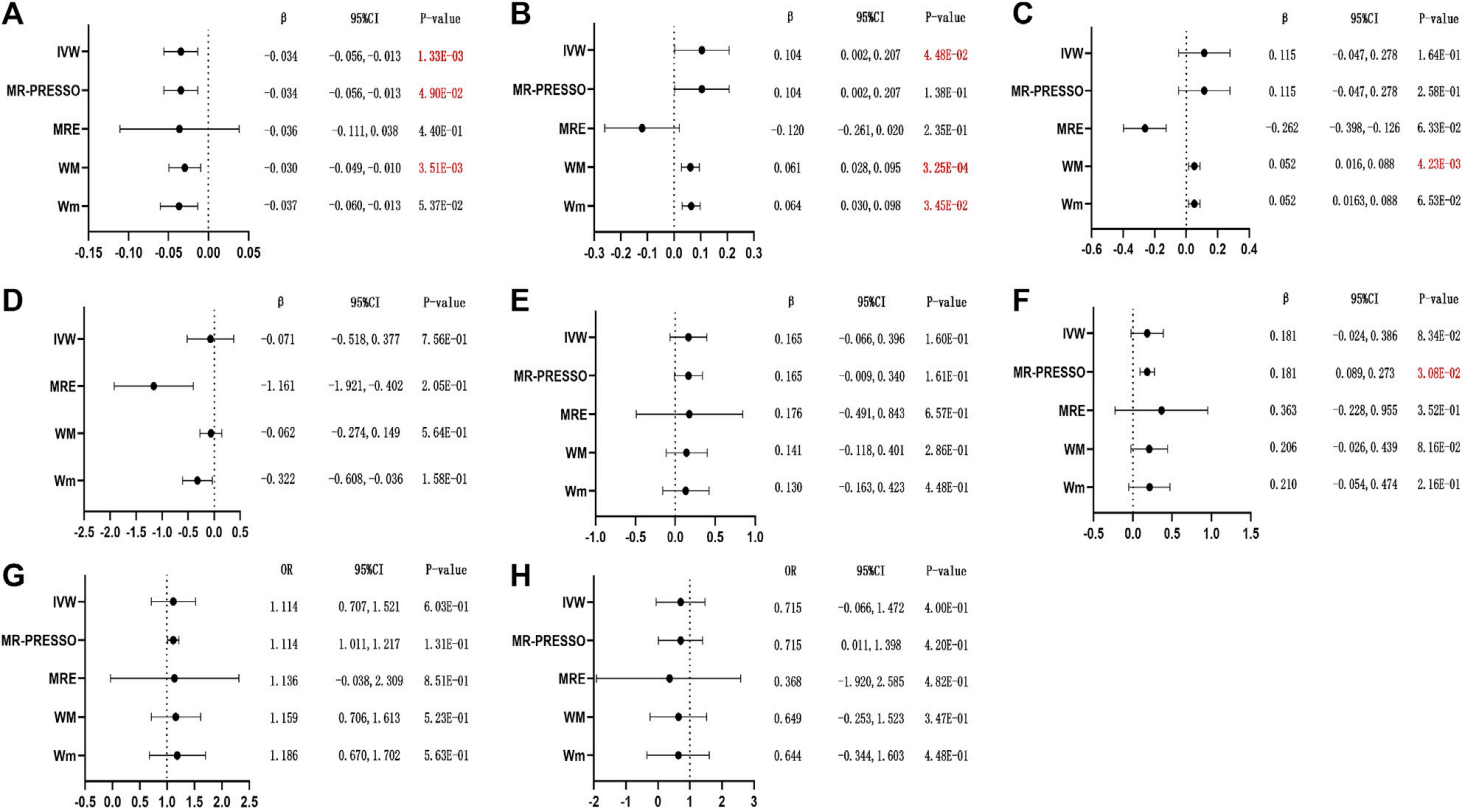
MR results of causal effects of coffee consumption SNPs on related outcome risks. (The estimates were derived from the random-effects IVW model, the MR-PRESSO approach, MRE regression, WM, and Wm. **(A–H)**: **(A)** SHBG; **(B)** bio-T; **(C)** TT; **(D)** E_2_; **(E)** AMH; **(F)** age at menopause; **(G)** irregular menstrual cycle/bleeding; and **(H)** dysmenorrhea. Outliers of MR-PRESSO: none or not available. *p*-values < 5.00E-02 are presented in red, and red and bold font means *p*-values < 2.08E-03. β, beta; CI, confidence interval; OR, odds ratio).

Although MR-PRESSO revealed suggestive evidence of an inverse association (*β*_MR-PRESSO_ = −0.034; 95% CI = −0.056, −0.013; *p* = 4.90E-02; Figure 3A), the intercept of MR-Egger and MR-PRESSO did not show significant directional pleiotropy (Supplementary Table S2). The random-effects IVW result supported that coffee consumption in women was significantly and inversely associated with their SHBG concentrations (corrected *β*_IVW_ = −0.034; 95% CI = −0.056, −0.013; *p* = 1.33E-03; Figure 3A). The weighted median approach led to a similar conclusion (*β*_WM_ = −0.030; 95% CI = −0.049, −0.010; *p* = 3.51E-03; Figure 3A), whereas the rest of the methods revealed no significant association between female coffee consumption and SHBG levels (Figure 3A).

Moreover, the result of the IVW analysis showed that coffee consumption in women exhibited a positive causal effect on bio-T levels (*β*_IVW_ = 0.104; 95% CI = 0.002, 0.207; *p* = 4.48E-02; Figure 3B), but the corrected result seemed to be not significant for MR-PRESSO (*p* = 1.38E-01; Figure 3B). There was a more significant association in the weighted median and weighted mode approach (*β*_WM_ = 0.061; 95% CI = 0.028, 0.095; *p* = 3.25E-04 and *β*_Wm_ = 0.064; 95% CI = 0.030, 0.098; *p* = 3.45E-02; Figure 3B). In addition, IVW analysis showed no association of genetically predicted female coffee drinkers with earlier age at menopause, while coffee drinking demonstrated a suggestive forward effect on age at menopause in women by MR-PRESSO (*β*_MR-PRESSO_ = 0.181; 95% CI = 0.089, 0.273; *p* = 3.08E-02; Figure 3F). Meanwhile, the directional pleiotropy of MR-PRESSO and MR-Egger did not show a significant difference (Supplementary Table S2). However, our analysis did not provide any evidence of a causal effect of female coffee consumption on their levels of TT, E2, and AMH (Figures 3C–E, respectively) or on the incidence of irregular menstrual cycle/bleeding and dysmenorrhea (Figures 3G, H).

Due to the heterogeneity present, we analyzed the results using the leave-one-out method and presented them in plots (Supplementary Figures S1–S3). Furthermore, the scatter plots, forest plots, and funnel plots were used to help interpret the results and assess potential sources of bias, and these plots are shown in Supplementary Figures S4–S12, respectively.

## 4 Discussion

Using summary data from large GWAS, our MR study revealed a significant association between smoking and female health, particularly with reproductive traits and abnormal menstrual phenotypes. The study found that smoking was significantly linked to an increasing risk of lower SHBG levels and earlier menopause, as well as a potential positive relationship with serum TT levels in women. In addition, there was suggestive evidence that alcohol consumption was associated with a higher risk of female higher TT levels and earlier menopause. Moreover, our study suggested that a potential effect of coffee consumption on women’s decreased SHBG levels. To conclude, our MR study indicated a significant causal relationship between these three modifiable risk factors and female reproductive traits and abnormal menstrual phenotypes based on hereditary information.

### 4.1 Smoking

The consistency of our MR findings with those from a large number of epidemiologic studies and smoking-by-gene interaction research studies supported a strongly causal association between female smokers and lower SHBG levels and earlier age at menopause and a potential effect of smoking on higher TT levels in female populations.

As it is well known, SHBG played a critical role in the transportation of sex hormones and regulation of their bioavailability. Furthermore, as reported (Wang, 2021), there was a link between lower SHBG levels and an increased incidence or prevalence of physical diseases, including hypertension, coronary heart disease, type 2 diabetes, PCOS, inflammation, and cancers. Therefore, investigating the condition of low SHBG was of utmost importance. Although several observational studies (Li et al., 2018; Soares et al., 2020; Qian et al., 2021; Wang, 2021; Jacobsen et al., 2022) have identified a negative correlation between smoking and SHBG levels in women, none of them have confirmed a causal relationship. Wang (2021) suggested a significant association of smoking with a 1.43-fold and 1.44-fold higher risk of low SHBG in women after adjusting for all tested confounders (*p* < 0.001). Soares et al. (2020) stated that smokers had lower SHBG levels than non-smokers during their reproductive age, but the causal relationship between smoking and the low SHBG concentration in women could not be established due to the cross-sectional nature of these studies, even if related confounders of the association were adjusted. A large randomized controlled trial of 1,000 female participants by Li et al. (2018) found that women exposed to second-hand smoke had high TT levels (*p* = 0.01), free androgen index (*p* = 0.001), and low SHBG levels (*p* = 0.03) in serum, compared to those in non-exposed women. Our research, using TSMR analysis, identified for the first time a significant inverse causal relationship between smoking and SHBG levels (*p* < 0.001). The result was consistent with the sensitivity analysis of IVW, MR-PRESSO, and WM, which provided strong evidence for the causal relationship. The failure of MRE might suggest outliers or violations of the inside assumptions in the cases of smoking initiation SNPs and the SHBG levels, while the MR-PRESSO result stated their causality relationship after correcting the outliers. Furthermore, the sensitivity analysis of the leave-one-out result also demonstrated robust evidence of their negative causality. Recently, a systematic review and meta-analysis of MR studies by Larsson and Burgess (2022) found that smoking women had disordered endocrine hormone levels, including SHBG and TT. Moreover, our study of MR-PRESSO and WM also observed a potential negative association between smoking and TT, consistent with previous meta-analysis and observational studies (Sowers et al., 2001; Barbieri et al., 2005; Zhao et al., 2016). Though a positive effect of smoking on the bio-T levels was found in the IVW and MR-PRESSO analyses, other methods did not produce congruent sensitive results. Therefore, the causal conclusion for bio-T was not clear and robust, and more sufficient studies and evidence were needed to support and supplement it. Finally, our study found no obvious detrimental effects of smoking on E_2_ and AMH levels in women (*p* > 0.05), consistent with the results of Dafopoulos’s multiple stepwise linear regression analysis (Dafopoulos et al., 2010).

Several recent studies have examined the association between smoking risk and earlier age at menopause. Mikkelsen et al. (2007) and a cohort study by (Tawfik et al. (2015) both considered that smoking was linked to a higher risk of early menopause, even after adjusting for age, site, and other confounders. A gene interaction study by Huang et al. (2022), less likely to be biased, confounded, or influenced by the environment and disease processes, showed that smoking alleles were associated with an increased risk of early menopause in East Asian women, suggesting that smoking may be involved in the etiology of menopause. However, no similar gene reports existed for European populations. A previous MR analysis (Ding et al., 2020) used two SNPs as IVs in the UK Biobank and found no causal association between menstrual flow and early menopause, but this study did not include the exposure phenotype of smoking initiation (whether smoking regularly) that we focused on, whereas our MR study using 314 smoking initiation SNPs as IVs proved a forward causality between smoking initiation and early menopause by IVW, MR-PRESSO, and WM methods. Several studies (Lutterodt et al., 2009; Tawfik et al., 2015; Hyland et al., 2016) suggested that byproducts found in cigarettes might have an adverse impact on follicle formation and hormone production, leading to early menopause. IVW and MR-PRESSO analyses also provided suggestive evidence that smoking women might be more likely to suffer from irregular menstrual cycle/bleeding and dysmenorrhea. However, no more methods could support this conclusion. A system review conducted 122 studies on 64,286 women to affirm that smoking women were associated with dysmenorrhea (Latthe et al., 2006), and an internet-based prospective cohort study by Hahn et al. (2013) revealed smoking might cause an increased prevalence of heavy menstrual bleeding and short menstrual cycles among women. However, in these previous studies, the exposure phenotype was just generally considered to be smoking, without subdividing into categories such as smoking initiation, current smoking, and smoking cessation. Therefore, more detailed and effective evidence was still required to support our conclusion on its causality.

### 4.2 Alcohol drinking

The relationship between alcohol and sex hormones levels has been uncertain in numerable previous research studies. A system review by Erol et al. (2019) suggested alcohol had an inverse correlation of SHBG and no obvious effect on TT. However, the available summary statistics for these two traits were primarily derived from studies conducted on male populations, rather than female populations. La Grange et al. (1995) proved that higher TT levels in young female adults were associated with higher alcohol consumption. However, no significant relationships were found between alcohol consumption and AMH in a cross-sectional study involving 477 women (Kline et al., 2016), nor with E_2_ based on UK Biobank data of premenopausal women (Tin Tin et al., 2021). Our MR study also found no correlation between alcohol consumption and serum levels of E_2_, SHBG, bio-T, and AMH, except for a suggestive causal effect of the alcohol drinking on TT levels. This conclusion was consistent with most preceding observational studies about female drinkers. Additionally, our MR study indicated that alcohol drinking might potentially cause early menopause, which was in agreement with previous surveys (Kinney et al., 2006; Choi et al., 2017). However, there was still no consensus on the relationship between drinking and irregular menstrual cycle/bleeding and dysmenorrhea. A cross-sectional study by Hahn et al. (2013) reported that alcohol consumption appeared to be related to an increased prevalence of irregular menstrual cycle and abnormal menstrual flow in women, while another comprehensive review by Onieva-Zafra et al. (2020) stated alcohol consumption had no causality with dysmenorrhea. Although the two studies were available and meaningful, they still had some limitations. For instance, they were restricted to a single population with small sample sizes, with the former focusing on 2,613 Danish participants and the latter on 311 Spanish participants. Therefore, their results did not provide adequate representation of the European female population. In our study, there was no significant association between alcohol and the abnormal menstrual phenotypes of irregular menstrual cycle/bleeding and dysmenorrhea. Therefore, further research was needed to fully understand the complex interactions between alcohol consumption and menstrual health.

### 4.3 Coffee consumption

Similarly, there remains a controversy in the relationship between coffee consumption and female serum sex hormone levels. A cross-sectional study by Pihan-Le Bars et al. (2017) demonstrated that coffee consumers had a reduced risk of low concentration of SHBG, particularly among postmenopausal women. Similar results (Goto et al., 2011; Goto et al., 2014) were observed in most studies between coffee and SHBG among postmenopausal women. While in premenopausal women with caffeine intake less than 371 mg/day, there was a decreasing trend of SHBG concentration with increased coffee consumption, although it was not significantly different (Kotsopoulos et al., 2009). This study also showed that there was no association between coffee consumption and testosterone and free testosterone. However, a limitation of this study was its sample size of only about 500 premenopausal women and being conducted in a single center. In our MR analysis, coffee had a potentially inverse association with SHBG concentration, while we could not distinguish SHBG concentration in premenopausal women from that in postmenopausal women due to the lack of raw data. Only few evidence existed to support the causal relationship between coffee and E_2_ (Schliep et al., 2016) and AMH concentrations (Kline et al., 2016). Meanwhile, no significant relationship was found between serum caffeine and the TT: E_2_ ratio (Schliep et al., 2016). Our MR analysis stated no robust evidence for the causality of coffee and the bio-T, TT, E_2_, and AMH levels, which was in accordance with the former reports.

Furthermore, our MR analysis yielded the same results as previous surveys (Kinney et al., 2006; Mikkelsen et al., 2007), concluding that coffee consumption did not affect age at menopause. Irregular menstrual cycle/bleeding and dysmenorrhea were considered the main abnormal menstrual phenotypes that might influence female reproduction. However, limited and controversial evidence existed regarding the correlation between coffee consumption and irregular menstrual cycle/bleeding and dysmenorrhea. For example, caffeine intake was not strongly related to an increased risk for menstrual cycle (Fenster et al., 1999). However, another prospective cohort study of women (Hahn et al., 2013) reported that coffee drinkers had a higher proportion of patients with irregular cycles, duration, and amount of menstrual flow. The main limitations of the studies were the lack of a defined normal reference for outcomes of the menstrual cycle period and menstrual bleeding, and outcome misclassification was also a concern for reports of duration of menstrual bleeding and heaviness of flow. Because some studies found that when women reported heavy blood loss, their flow was actually within the normal range as determined by the measurement of actual blood loss collected from pads and tampons. Actually, we did not identify any causal effects of coffee on these two diseases based on our TSMR methods.

To sum up, based on the results of our analyses and relevant studies, further research on the underlying mechanisms between smoking, alcohol and coffee consumption, and female reproductive health was warranted.

## 5 Strengths and limitations

Our MR study had several key strengths, such as assessing the impact of three lifestyle behaviors on female reproductive health, including the reproduction-related hormones and menstrual phenotypes. We also used exposure–outcome data from large and different consortia, which improved the reliability of our results and eliminated overlapping. Furthermore, our study was based on the law of independent assortment, which largely avoided potential bias due to artificial errors, confounding, and reverse causation. However, there were inevitably some limitations to our study. First, the GWAS data used in our study were only from European individuals, whose results were not representative of other races or geographic areas. Second, the datasets of these three lifestyle behaviors performed contained both male and female populations, which might introduce collider bias. Third, we did not stratify the related hormones by age, which could vary significantly between premenopausal and postmenopausal women.

## 6 Conclusion and future research

To our knowledge, this study was the first research using MR analysis to address the possible causal correlation of smoking, alcohol drinking, and coffee consumption with female reproductive health, especially focusing on sex hormones and abnormal menstrual phenotypes. Our study affirmed that smoking played an important role in reducing SHBG levels and advancing menopause in women. Moreover, female alcohol drinkers might lead to higher TT levels and earlier menopause, while coffee consumption potentially affected the decrease in SHBG levels. However, further research is necessary to distinguish the genetic instruments for these exposures between male and female populations, to differentiate the outcomes for premenopausal and postmenopausal women, and to investigate the generalizability of these findings in non-European populations.

## Data Availability

The original contributions presented in the study are included in the article/Supplementary Material; further inquiries can be directed to the corresponding authors.
