# Supplementary material for: The causal association between smoking initiation, alcohol and coffee consumption, and women’s reproductive health: A two-sample Mendelian randomization analysis
Source: Front Genet. 2023 Apr 6;14:1098616. doi: 10.3389/fgene.2023.1098616 (PMC10117654; doi:10.3389/fgene.2023.1098616)
Supplement: Supplementary file 1 [file DataSheet1.ZIP › Supplementary_Materials/Supplementary_Material.docx]

Supplementary Material

Supplementary Table 1. The characteristics of smoking initiation, alcohol drinking and coffee consumption associated SNPs used in our MR study

| **Phenotype** | **SNP** | **Chromosome:Position** | **Ethnicity** | **Beta** | **Standard Error** | **P-value** | **Effect_allele/Other_allele** | **Effect allele frequency** | **R2** | **F Statistic** |
| --- | --- | --- | --- | --- | --- | --- | --- | --- | --- | --- |
| SmokingInitiation | rs10042827 | 5:170299916 | European | 0.0167 | 0.0027 | 9.41E-10 | C/T | 0.6810 | 3.04E-05 | 37.40 |
| SmokingInitiation | rs1004787 | 2:45159091 | European | 0.0284 | 0.0026 | 1.11E-28 | A/G | 0.5520 | 9.98E-05 | 123.00 |
| SmokingInitiation | rs10060196 | 5:106455988 | European | 0.0183 | 0.0026 | 1.29E-12 | A/C | 0.5806 | 4.08E-05 | 50.30 |
| SmokingInitiation | rs1008078 | 1:91189731 | European | 0.0228 | 0.0026 | 1.63E-18 | T/C | 0.4020 | 6.26E-05 | 77.10 |
| SmokingInitiation | rs1022376 | 2:22067213 | European | -0.0147 | 0.0026 | 1.66E-08 | C/T | 0.5158 | 2.71E-05 | 31.90 |
| SmokingInitiation | rs1022528 | 1:71490122 | European | 0.0174 | 0.0027 | 8.48E-11 | A/G | 0.3440 | 3.42E-05 | 42.10 |
| SmokingInitiation | rs10233018 | 7:117523709 | European | 0.0246 | 0.0025 | 4.77E-22 | G/A | 0.5160 | 7.56E-05 | 93.20 |
| SmokingInitiation | rs10259715 | 7:3329967 | European | -0.0187 | 0.0032 | 6.42E-09 | A/T | 0.2099 | 2.89E-05 | 33.70 |
| SmokingInitiation | rs10446419 | 3:25725501 | European | -0.0196 | 0.0031 | 5.05E-10 | G/A | 0.2070 | 3.14E-05 | 38.70 |
| SmokingInitiation | rs10490159 | 2:51341259 | European | 0.0172 | 0.0026 | 3.86E-11 | T/C | 0.3940 | 3.55E-05 | 43.70 |
| SmokingInitiation | rs1050847 | 16:87443734 | European | -0.0148 | 0.0026 | 7.37E-09 | T/C | 0.5590 | 2.71E-05 | 33.40 |
| SmokingInitiation | rs10698713 | 6:158882320 | European | -0.0335 | 0.0056 | 2.38E-09 | A/G | 0.0544 | 2.89E-05 | 35.60 |
| SmokingInitiation | rs10789369 | 1:73824909 | European | -0.0234 | 0.0026 | 3.39E-19 | G/A | 0.6150 | 6.51E-05 | 80.20 |
| SmokingInitiation | rs10805858 | 5:88873832 | European | 0.0181 | 0.0027 | 1.88E-11 | T/A | 0.3353 | 3.66E-05 | 45.10 |
| SmokingInitiation | rs10853981 | 19:4965064 | European | 0.0148 | 0.0027 | 4.88E-08 | A/G | 0.3304 | 2.42E-05 | 29.80 |
| SmokingInitiation | rs10858334 | 9:137989785 | European | 0.0229 | 0.0038 | 1.18E-09 | G/C | 0.1400 | 3.15E-05 | 37.00 |
| SmokingInitiation | rs10873871 | 1:76689019 | European | 0.0175 | 0.0031 | 2.82E-08 | G/A | 0.2070 | 2.50E-05 | 30.80 |
| SmokingInitiation | rs10885480 | 10:115378364 | European | -0.0187 | 0.0028 | 3.83E-11 | C/T | 0.2840 | 3.55E-05 | 43.70 |
| SmokingInitiation | rs10905461 | 10:8803551 | European | -0.0164 | 0.0029 | 2.36E-08 | C/T | 0.7480 | 2.53E-05 | 31.20 |
| SmokingInitiation | rs10914684 | 1:33795572 | European | -0.0158 | 0.0027 | 6.32E-09 | A/G | 0.3240 | 2.74E-05 | 33.70 |
| SmokingInitiation | rs10935779 | 3:149543102 | European | -0.0143 | 0.0026 | 2.95E-08 | T/C | 0.4150 | 2.49E-05 | 30.70 |
| SmokingInitiation | rs10945141 | 6:69470709 | European | 0.0181 | 0.0029 | 3.59E-10 | A/G | 0.2630 | 3.19E-05 | 39.30 |
| SmokingInitiation | rs10953957 | 7:121954709 | European | 0.0144 | 0.0026 | 3.66E-08 | A/G | 0.3860 | 2.46E-05 | 30.30 |
| SmokingInitiation | rs10966092 | 9:23831658 | European | -0.0205 | 0.0029 | 1.12E-12 | C/T | 0.2670 | 4.11E-05 | 50.60 |
| SmokingInitiation | rs10969352 | 9:29747488 | European | 0.0143 | 0.0025 | 1.82E-08 | A/T | 0.5000 | 2.57E-05 | 31.70 |
| SmokingInitiation | rs11057005 | 12:16748721 | European | -0.0157 | 0.0026 | 9.12E-10 | G/A | 0.4410 | 3.04E-05 | 37.50 |
| SmokingInitiation | rs1106363 | 11:131966264 | European | 0.0174 | 0.0027 | 9.20E-11 | T/C | 0.3446 | 3.41E-05 | 42.00 |
| SmokingInitiation | rs11076962 | 16:5811367 | European | 0.0183 | 0.0028 | 1.20E-10 | C/T | 0.2790 | 3.37E-05 | 41.50 |
| SmokingInitiation | rs1108130 | 13:100648356 | European | 0.0239 | 0.0031 | 1.57E-14 | A/T | 0.2120 | 4.79E-05 | 59.00 |
| SmokingInitiation | rs1109480 | 12:121083279 | European | -0.0167 | 0.0026 | 1.84E-10 | A/G | 0.3840 | 3.30E-05 | 40.60 |
| SmokingInitiation | rs11128203 | 3:71064431 | European | 0.0204 | 0.0026 | 1.29E-15 | A/T | 0.5300 | 5.19E-05 | 63.90 |
| SmokingInitiation | rs11162019 | 1:87913176 | European | -0.0155 | 0.0026 | 5.06E-09 | T/C | 0.3630 | 2.78E-05 | 34.20 |
| SmokingInitiation | rs1116690 | 4:143510148 | European | 0.0163 | 0.0029 | 2.16E-08 | G/A | 0.7420 | 2.54E-05 | 31.30 |
| SmokingInitiation | rs11192347 | 10:106929313 | European | -0.0265 | 0.0043 | 6.15E-10 | A/G | 0.1040 | 3.26E-05 | 38.30 |
| SmokingInitiation | rs11258417 | 10:13533053 | European | -0.0145 | 0.0026 | 2.71E-08 | T/C | 0.3910 | 2.51E-05 | 30.90 |
| SmokingInitiation | rs1126757 | 19:55879872 | European | 0.0142 | 0.0026 | 2.92E-08 | T/C | 0.4730 | 2.50E-05 | 30.80 |
| SmokingInitiation | rs112725451 | 4:68017710 | European | 0.0261 | 0.0034 | 1.65E-14 | T/C | 0.1690 | 4.78E-05 | 58.90 |
| SmokingInitiation | rs113230003 | 19:18460956 | European | -0.0189 | 0.0029 | 1.05E-10 | A/G | 0.2550 | 3.38E-05 | 41.70 |
| SmokingInitiation | rs1139897 | 16:720986 | European | -0.0241 | 0.0030 | 1.77E-15 | A/G | 0.2300 | 5.14E-05 | 63.30 |
| SmokingInitiation | rs114976176 | 2:264621 | European | -0.0155 | 0.0027 | 6.04E-09 | C/A | 0.3516 | 2.74E-05 | 33.80 |
| SmokingInitiation | rs1150668 | 6:28129789 | European | -0.0185 | 0.0026 | 8.54E-13 | G/T | 0.4190 | 4.17E-05 | 51.20 |
| SmokingInitiation | rs11594623 | 10:103960351 | European | 0.0274 | 0.0030 | 7.45E-20 | C/T | 0.2342 | 6.75E-05 | 83.20 |
| SmokingInitiation | rs1160685 | 4:94052854 | European | 0.0153 | 0.0026 | 2.31E-09 | G/C | 0.4500 | 2.90E-05 | 35.70 |
| SmokingInitiation | rs11611651 | 12:133380790 | European | 0.0271 | 0.0045 | 2.05E-09 | A/G | 0.0868 | 2.91E-05 | 35.90 |
| SmokingInitiation | rs11642231 | 16:89608702 | European | -0.0156 | 0.0026 | 3.44E-09 | A/G | 0.3690 | 2.83E-05 | 34.90 |
| SmokingInitiation | rs11651955 | 17:16235462 | European | -0.0140 | 0.0025 | 3.74E-08 | A/G | 0.4990 | 2.46E-05 | 30.30 |
| SmokingInitiation | rs11692435 | 2:98275354 | European | 0.0251 | 0.0046 | 4.47E-08 | A/G | 0.0848 | 2.44E-05 | 29.90 |
| SmokingInitiation | rs11713899 | 3:2365026 | European | 0.0187 | 0.0034 | 3.15E-08 | C/A | 0.1710 | 2.48E-05 | 30.60 |
| SmokingInitiation | rs1173461 | 5:157707571 | European | 0.0166 | 0.0027 | 9.51E-10 | T/C | 0.3270 | 3.04E-05 | 37.40 |
| SmokingInitiation | rs117657830 | 16:75766873 | European | -0.0378 | 0.0064 | 3.18E-09 | G/A | 0.0417 | 2.85E-05 | 35.10 |
| SmokingInitiation | rs11766326 | 7:111100585 | European | -0.0175 | 0.0026 | 1.79E-11 | C/T | 0.5060 | 3.85E-05 | 45.20 |
| SmokingInitiation | rs11768481 | 7:96629103 | European | -0.0186 | 0.0027 | 5.23E-12 | A/C | 0.3400 | 3.86E-05 | 47.60 |
| SmokingInitiation | rs117734003 | 19:51129745 | European | 0.0303 | 0.0051 | 2.57E-09 | C/G | 0.0673 | 2.88E-05 | 35.50 |
| SmokingInitiation | rs11783093 | 8:27425349 | European | -0.0471 | 0.0035 | 2.07E-41 | T/C | 0.1580 | 1.48E-04 | 182.00 |
| SmokingInitiation | rs11791671 | 9:3398679 | European | 0.0279 | 0.0051 | 4.24E-08 | T/C | 0.0673 | 2.43E-05 | 30.00 |
| SmokingInitiation | rs118202 | 6:111658371 | European | -0.0367 | 0.0033 | 1.90E-29 | T/G | 0.8120 | 1.03E-04 | 127.00 |
| SmokingInitiation | rs11872397 | 18:72535282 | European | -0.0171 | 0.0029 | 5.20E-09 | A/G | 0.2530 | 2.77E-05 | 34.10 |
| SmokingInitiation | rs1187820 | 3:173072584 | European | -0.0143 | 0.0026 | 2.69E-08 | T/C | 0.4390 | 2.51E-05 | 30.90 |
| SmokingInitiation | rs11956866 | 5:161018271 | European | -0.0148 | 0.0026 | 7.82E-09 | G/T | 0.5670 | 2.70E-05 | 33.30 |
| SmokingInitiation | rs12022778 | 1:50603995 | European | 0.0268 | 0.0032 | 3.18E-17 | C/A | 0.2020 | 5.80E-05 | 71.20 |
| SmokingInitiation | rs12027999 | 1:154206358 | European | -0.0244 | 0.0039 | 5.33E-10 | C/T | 0.1200 | 3.13E-05 | 38.60 |
| SmokingInitiation | rs12130857 | 1:7791461 | European | -0.0180 | 0.0027 | 3.65E-11 | A/G | 0.3250 | 3.55E-05 | 43.80 |
| SmokingInitiation | rs12195240 | 6:98636905 | European | 0.0249 | 0.0028 | 1.08E-18 | A/G | 0.2850 | 6.32E-05 | 77.90 |
| SmokingInitiation | rs12244388 | 10:104640052 | European | 0.0258 | 0.0027 | 4.31E-22 | A/G | 0.3500 | 7.58E-05 | 93.40 |
| SmokingInitiation | rs12474587 | 2:162802993 | European | 0.0242 | 0.0026 | 4.83E-21 | T/G | 0.4290 | 7.19E-05 | 88.60 |
| SmokingInitiation | rs12517438 | 5:30842054 | European | 0.0154 | 0.0026 | 1.89E-09 | G/T | 0.5380 | 2.93E-05 | 36.10 |
| SmokingInitiation | rs12563365 | 1:236872829 | European | 0.0166 | 0.0026 | 1.05E-10 | A/G | 0.5560 | 3.38E-05 | 41.70 |
| SmokingInitiation | rs12633090 | 3:83241365 | European | -0.0230 | 0.0033 | 3.16E-12 | C/G | 0.1820 | 3.94E-05 | 48.60 |
| SmokingInitiation | rs12714017 | 2:80999398 | European | 0.0154 | 0.0026 | 3.65E-09 | C/T | 0.5110 | 2.96E-05 | 34.80 |
| SmokingInitiation | rs12739243 | 1:210302043 | European | -0.0213 | 0.0031 | 4.45E-12 | C/T | 0.2210 | 3.89E-05 | 47.90 |
| SmokingInitiation | rs12740789 | 1:72752073 | European | -0.0285 | 0.0033 | 1.18E-17 | A/G | 0.1780 | 5.94E-05 | 73.20 |
| SmokingInitiation | rs12755632 | 1:41776623 | European | -0.0154 | 0.0027 | 1.93E-08 | G/A | 0.3160 | 2.56E-05 | 31.60 |
| SmokingInitiation | rs12855717 | 13:101252635 | European | 0.0155 | 0.0026 | 1.22E-09 | T/C | 0.5380 | 2.99E-05 | 36.90 |
| SmokingInitiation | rs12878369 | 14:28346502 | European | 0.0174 | 0.0026 | 1.60E-11 | A/C | 0.4148 | 3.69E-05 | 45.40 |
| SmokingInitiation | rs12918191 | 16:50945156 | European | -0.0197 | 0.0030 | 3.14E-11 | G/A | 0.2430 | 3.58E-05 | 44.10 |
| SmokingInitiation | rs1291821 | 10:11133823 | European | 0.0145 | 0.0026 | 1.39E-08 | G/A | 0.5340 | 2.61E-05 | 32.20 |
| SmokingInitiation | rs13007361 | 2:166250244 | European | 0.0175 | 0.0031 | 2.29E-08 | A/G | 0.2080 | 2.53E-05 | 31.20 |
| SmokingInitiation | rs13066050 | 3:81325861 | European | 0.0188 | 0.0031 | 1.93E-09 | T/C | 0.2080 | 2.92E-05 | 36.00 |
| SmokingInitiation | rs13109980 | 4:140886963 | European | -0.0222 | 0.0027 | 3.37E-16 | A/G | 0.3260 | 5.41E-05 | 66.60 |
| SmokingInitiation | rs13110073 | 4:147797913 | European | -0.0246 | 0.0026 | 3.24E-21 | C/T | 0.3950 | 7.26E-05 | 89.40 |
| SmokingInitiation | rs13237637 | 7:3503207 | European | -0.0237 | 0.0025 | 1.54E-20 | C/G | 0.4850 | 7.00E-05 | 86.30 |
| SmokingInitiation | rs13261666 | 8:59814666 | European | -0.0200 | 0.0025 | 4.36E-15 | T/G | 0.5170 | 4.99E-05 | 61.50 |
| SmokingInitiation | rs13392222 | 2:100672408 | European | -0.0234 | 0.0037 | 1.93E-10 | C/A | 0.1390 | 3.29E-05 | 40.50 |
| SmokingInitiation | rs13437771 | 7:99071478 | European | -0.0271 | 0.0035 | 1.39E-14 | G/A | 0.1550 | 4.81E-05 | 59.30 |
| SmokingInitiation | rs1373178 | 18:49967811 | European | -0.0203 | 0.0026 | 4.16E-15 | G/T | 0.5880 | 5.00E-05 | 61.60 |
| SmokingInitiation | rs1381287 | 14:98597552 | European | 0.0180 | 0.0026 | 1.81E-12 | T/C | 0.4670 | 4.04E-05 | 49.70 |
| SmokingInitiation | rs1381775 | 11:42442826 | European | -0.0156 | 0.0028 | 2.79E-08 | C/T | 0.7120 | 2.50E-05 | 30.80 |
| SmokingInitiation | rs1385108 | 5:154839646 | European | 0.0187 | 0.0030 | 3.84E-10 | T/C | 0.2390 | 3.18E-05 | 39.20 |
| SmokingInitiation | rs13906 | 12:49952394 | European | -0.0245 | 0.0041 | 1.98E-09 | T/C | 0.1090 | 2.92E-05 | 36.00 |
| SmokingInitiation | rs1435479 | 4:94550450 | European | 0.0164 | 0.0028 | 5.68E-09 | T/G | 0.2875 | 2.75E-05 | 33.90 |
| SmokingInitiation | rs1435672 | 15:36399479 | European | 0.0141 | 0.0026 | 3.82E-08 | C/T | 0.5600 | 2.45E-05 | 30.20 |
| SmokingInitiation | rs1445649 | 2:155682556 | European | 0.0206 | 0.0026 | 8.48E-16 | C/T | 0.5380 | 5.26E-05 | 64.80 |
| SmokingInitiation | rs1449012 | 3:159048333 | European | -0.0154 | 0.0026 | 1.77E-09 | T/C | 0.4630 | 2.94E-05 | 36.20 |
| SmokingInitiation | rs147052174 | 1:179783167 | European | 0.0623 | 0.0098 | 2.30E-10 | T/G | 0.0171 | 3.26E-05 | 40.20 |
| SmokingInitiation | rs1514176 | 1:74991596 | European | -0.0193 | 0.0026 | 7.67E-14 | A/G | 0.5800 | 4.54E-05 | 55.90 |
| SmokingInitiation | rs1518393 | 2:58171220 | European | 0.0169 | 0.0026 | 1.30E-10 | C/A | 0.6190 | 3.35E-05 | 41.30 |
| SmokingInitiation | rs1549979 | 3:85460131 | European | -0.0245 | 0.0026 | 8.80E-21 | T/C | 0.6150 | 7.12E-05 | 87.40 |
| SmokingInitiation | rs160631 | 6:52895230 | European | -0.0173 | 0.0029 | 1.87E-09 | G/T | 0.7310 | 2.93E-05 | 36.10 |
| SmokingInitiation | rs1632941 | 6:29796685 | European | -0.0158 | 0.0026 | 6.67E-10 | C/T | 0.4600 | 3.10E-05 | 38.10 |
| SmokingInitiation | rs16826827 | 2:147825689 | European | -0.0222 | 0.0039 | 9.17E-09 | C/T | 0.1240 | 2.68E-05 | 33.00 |
| SmokingInitiation | rs16828799 | 3:173353739 | European | 0.0198 | 0.0035 | 1.83E-08 | T/G | 0.1560 | 2.57E-05 | 31.70 |
| SmokingInitiation | rs1713676 | 11:113660576 | European | -0.0167 | 0.0026 | 5.38E-11 | G/A | 0.5225 | 3.49E-05 | 43.00 |
| SmokingInitiation | rs1714521 | 3:158284861 | European | -0.0163 | 0.0026 | 3.07E-10 | C/A | 0.4110 | 3.21E-05 | 39.60 |
| SmokingInitiation | rs17165769 | 5:107365642 | European | 0.0159 | 0.0026 | 9.56E-10 | G/A | 0.3949 | 3.04E-05 | 37.40 |
| SmokingInitiation | rs17197663 | 13:38172867 | European | -0.0216 | 0.0039 | 2.06E-08 | A/G | 0.1250 | 2.55E-05 | 31.40 |
| SmokingInitiation | rs17229285 | 2:199523122 | European | -0.0155 | 0.0025 | 1.27E-09 | T/C | 0.5050 | 2.99E-05 | 36.90 |
| SmokingInitiation | rs1733760 | 10:56698174 | European | 0.0148 | 0.0025 | 6.70E-09 | C/T | 0.5100 | 2.73E-05 | 33.60 |
| SmokingInitiation | rs1737329 | 6:163807748 | European | 0.0170 | 0.0029 | 5.08E-09 | G/C | 0.7420 | 2.78E-05 | 34.20 |
| SmokingInitiation | rs17554906 | 6:92226609 | European | 0.0142 | 0.0026 | 3.14E-08 | C/G | 0.4440 | 2.48E-05 | 30.60 |
| SmokingInitiation | rs1759433 | 9:128073097 | European | 0.0154 | 0.0026 | 1.69E-09 | A/G | 0.4800 | 2.95E-05 | 36.30 |
| SmokingInitiation | rs17616642 | 2:59022210 | European | -0.0166 | 0.0030 | 0.000000021 | G/A | 0.2469 | 2.55E-05 | 31.40 |
| SmokingInitiation | rs17692129 | 17:44793283 | European | 0.0196 | 0.0027 | 4.57E-13 | T/C | 0.3310 | 4.25E-05 | 52.40 |
| SmokingInitiation | rs1772572 | 13:81191176 | European | -0.0169 | 0.0027 | 5.62E-10 | A/C | 0.3241 | 3.12E-05 | 38.40 |
| SmokingInitiation | rs1799068 | 7:97707069 | European | 0.0166 | 0.0026 | 2.59E-10 | T/G | 0.3790 | 3.25E-05 | 40.00 |
| SmokingInitiation | rs1811739 | 14:77529375 | European | 0.0183 | 0.0030 | 5.97E-10 | A/G | 0.2480 | 3.11E-05 | 38.30 |
| SmokingInitiation | rs1863161 | 2:60139524 | European | 0.0153 | 0.0026 | 2.34E-09 | A/G | 0.5609 | 2.90E-05 | 35.70 |
| SmokingInitiation | rs1889571 | 1:32195819 | European | 0.0222 | 0.0038 | 4.19E-09 | G/T | 0.1310 | 2.80E-05 | 34.50 |
| SmokingInitiation | rs1901477 | 2:104126983 | European | 0.0304 | 0.0026 | 2.07E-31 | G/A | 0.5110 | 1.16E-04 | 136.00 |
| SmokingInitiation | rs1927901 | 9:120519111 | European | -0.0142 | 0.0026 | 0.000000031 | C/T | 0.5530 | 2.48E-05 | 30.60 |
| SmokingInitiation | rs1930371 | 9:81444104 | European | -0.0172 | 0.0030 | 7.09E-09 | T/C | 0.2410 | 2.72E-05 | 33.50 |
| SmokingInitiation | rs1931431 | 9:11161799 | European | 0.0182 | 0.0026 | 8.56E-13 | C/G | 0.4780 | 4.15E-05 | 51.10 |
| SmokingInitiation | rs1937443 | 1:66469643 | European | 0.0204 | 0.0026 | 1.79E-15 | G/C | 0.5630 | 5.14E-05 | 63.30 |
| SmokingInitiation | rs2010921 | 11:132098205 | European | 0.0174 | 0.0028 | 2.47E-10 | A/G | 0.3110 | 3.25E-05 | 40.10 |
| SmokingInitiation | rs2028269 | 5:79308315 | European | 0.0162 | 0.0026 | 5.19E-10 | A/G | 0.3990 | 3.13E-05 | 38.60 |
| SmokingInitiation | rs2063976 | 8:91096366 | European | -0.0202 | 0.0027 | 7.45E-14 | T/C | 0.6650 | 4.54E-05 | 55.90 |
| SmokingInitiation | rs2155646 | 11:112912811 | European | 0.0378 | 0.0026 | 9.44E-48 | C/T | 0.4000 | 1.71E-04 | 211.00 |
| SmokingInitiation | rs2173019 | 5:167614971 | European | 0.0282 | 0.0033 | 2.98E-17 | A/T | 0.1770 | 5.79E-05 | 71.40 |
| SmokingInitiation | rs221988 | 3:64234307 | European | -0.0149 | 0.0026 | 1.43E-08 | C/A | 0.3840 | 2.61E-05 | 32.20 |
| SmokingInitiation | rs2276825 | 3:52886605 | European | 0.0189 | 0.0030 | 1.89E-10 | C/T | 0.2450 | 3.30E-05 | 40.60 |
| SmokingInitiation | rs2279829 | 3:147106319 | European | -0.0174 | 0.0031 | 2.05E-08 | T/C | 0.2160 | 2.56E-05 | 31.50 |
| SmokingInitiation | rs2289791 | 15:67476952 | European | -0.0177 | 0.0030 | 2.01E-09 | T/G | 0.2470 | 2.92E-05 | 36.00 |
| SmokingInitiation | rs2306866 | 3:53766212 | European | -0.0167 | 0.0026 | 1.89E-10 | T/A | 0.6140 | 3.30E-05 | 40.60 |
| SmokingInitiation | rs2319545 | 3:147719648 | European | 0.0232 | 0.0036 | 8.30E-11 | A/C | 0.1491 | 3.42E-05 | 42.20 |
| SmokingInitiation | rs2344976 | 17:30685935 | European | -0.0151 | 0.0026 | 7.98E-09 | C/T | 0.6120 | 2.70E-05 | 33.30 |
| SmokingInitiation | rs2359180 | 18:41314171 | European | -0.0144 | 0.0026 | 4.98E-08 | G/A | 0.3690 | 2.41E-05 | 29.70 |
| SmokingInitiation | rs2378662 | 9:86707289 | European | 0.0152 | 0.0026 | 2.67E-09 | A/G | 0.5410 | 2.87E-05 | 35.40 |
| SmokingInitiation | rs238896 | 11:113994505 | European | -0.0169 | 0.0025 | 3.65E-11 | A/G | 0.4900 | 3.55E-05 | 43.80 |
| SmokingInitiation | rs2526390 | 3:50192760 | European | 0.0205 | 0.0027 | 3.62E-14 | T/C | 0.3340 | 4.66E-05 | 57.40 |
| SmokingInitiation | rs2539706 | 2:59819545 | European | 0.0162 | 0.0026 | 1.95E-10 | A/G | 0.5299 | 3.29E-05 | 40.50 |
| SmokingInitiation | rs2587507 | 17:77790135 | European | -0.0147 | 0.0025 | 8.69E-09 | C/T | 0.5020 | 2.69E-05 | 33.10 |
| SmokingInitiation | rs2637869 | 1:38757237 | European | 0.0182 | 0.0028 | 6.54E-11 | A/G | 0.2970 | 3.47E-05 | 42.70 |
| SmokingInitiation | rs2710634 | 2:32808804 | European | -0.0178 | 0.0026 | 3.36E-12 | C/T | 0.5210 | 3.94E-05 | 48.50 |
| SmokingInitiation | rs2734390 | 3:60459291 | European | 0.0148 | 0.0026 | 2.09E-08 | G/A | 0.3720 | 2.55E-05 | 31.40 |
| SmokingInitiation | rs2796793 | 10:36634124 | European | 0.0145 | 0.0026 | 1.55E-08 | A/G | 0.4520 | 2.60E-05 | 32.00 |
| SmokingInitiation | rs281296 | 15:47685010 | European | 0.0247 | 0.0027 | 1.59E-20 | A/G | 0.3570 | 7.00E-05 | 86.20 |
| SmokingInitiation | rs28408682 | 10:104403310 | European | 0.0167 | 0.0026 | 1.41E-10 | G/A | 0.6000 | 3.34E-05 | 41.10 |
| SmokingInitiation | rs28441558 | 17:7803118 | European | -0.0356 | 0.0055 | 1.24E-10 | C/T | 0.0563 | 3.36E-05 | 41.40 |
| SmokingInitiation | rs2901785 | 1:174104743 | European | -0.0173 | 0.0026 | 1.47E-11 | A/G | 0.4460 | 3.70E-05 | 45.60 |
| SmokingInitiation | rs290601 | 8:115374642 | European | 0.0163 | 0.0029 | 1.14E-08 | T/C | 0.2740 | 2.65E-05 | 32.60 |
| SmokingInitiation | rs2925128 | 14:98362355 | European | 0.0168 | 0.0027 | 3.67E-10 | T/C | 0.3852 | 3.35E-05 | 39.30 |
| SmokingInitiation | rs2939756 | 11:41436297 | European | -0.0157 | 0.0026 | 7.45E-10 | A/G | 0.4800 | 3.08E-05 | 37.90 |
| SmokingInitiation | rs2959084 | 11:46078656 | European | 0.0171 | 0.0028 | 9.82E-10 | A/G | 0.7047 | 3.04E-05 | 37.40 |
| SmokingInitiation | rs301807 | 1:8484823 | European | 0.0180 | 0.0026 | 2.50E-12 | G/A | 0.5700 | 3.98E-05 | 49.00 |
| SmokingInitiation | rs3115418 | 2:200936399 | European | -0.0142 | 0.0026 | 2.79E-08 | C/T | 0.4540 | 2.51E-05 | 30.90 |
| SmokingInitiation | rs3172494 | 3:48731487 | European | -0.0291 | 0.0040 | 3.40E-13 | T/G | 0.1150 | 4.32E-05 | 53.00 |
| SmokingInitiation | rs3218116 | 6:41901763 | European | -0.0198 | 0.0029 | 1.05E-11 | T/C | 0.2560 | 3.75E-05 | 46.20 |
| SmokingInitiation | rs329124 | 5:133865452 | European | -0.0164 | 0.0026 | 1.96E-10 | G/A | 0.4280 | 3.29E-05 | 40.50 |
| SmokingInitiation | rs34342129 | 18:5872472 | European | -0.0143 | 0.0025 | 2.13E-08 | C/T | 0.5090 | 2.55E-05 | 31.40 |
| SmokingInitiation | rs34399632 | 2:137571174 | European | 0.0194 | 0.0030 | 1.46E-10 | G/A | 0.2320 | 3.34E-05 | 41.10 |
| SmokingInitiation | rs34553878 | 9:134334588 | European | 0.0247 | 0.0041 | 1.17E-09 | G/A | 0.1110 | 3.00E-05 | 37.00 |
| SmokingInitiation | rs34940743 | 14:80102233 | European | 0.0159 | 0.0027 | 2.80E-09 | G/A | 0.3460 | 2.87E-05 | 35.30 |
| SmokingInitiation | rs35375873 | 5:43190647 | European | -0.0270 | 0.0041 | 3.29E-11 | C/G | 0.1100 | 3.57E-05 | 44.00 |
| SmokingInitiation | rs35656245 | 1:190957480 | European | 0.0159 | 0.0029 | 2.23E-08 | A/G | 0.2760 | 2.54E-05 | 31.30 |
| SmokingInitiation | rs357304 | 2:164862639 | European | 0.0167 | 0.0029 | 5.40E-09 | C/T | 0.7270 | 2.76E-05 | 34.00 |
| SmokingInitiation | rs359247 | 2:60477052 | European | 0.0220 | 0.0027 | 9.89E-17 | T/A | 0.6387 | 5.60E-05 | 69.00 |
| SmokingInitiation | rs359431 | 5:173288534 | European | -0.0142 | 0.0026 | 3.16E-08 | T/C | 0.5600 | 2.48E-05 | 30.60 |
| SmokingInitiation | rs3740977 | 11:46393574 | European | 0.0195 | 0.0034 | 1.17E-08 | C/T | 0.1670 | 2.64E-05 | 32.50 |
| SmokingInitiation | rs3800227 | 6:108994161 | European | 0.0172 | 0.0029 | 3.64E-09 | G/A | 0.7420 | 2.82E-05 | 34.80 |
| SmokingInitiation | rs3810496 | 20:62406886 | European | 0.0159 | 0.0026 | 1.54E-09 | C/T | 0.6194 | 2.97E-05 | 36.50 |
| SmokingInitiation | rs3811038 | 2:113240183 | European | 0.0191 | 0.0028 | 1.58E-11 | C/T | 0.2790 | 3.68E-05 | 45.40 |
| SmokingInitiation | rs3820277 | 1:18436657 | European | -0.0188 | 0.0026 | 1.57E-13 | T/G | 0.5260 | 4.42E-05 | 54.50 |
| SmokingInitiation | rs3843905 | 5:165427280 | European | -0.0151 | 0.0026 | 5.41E-09 | T/C | 0.4030 | 2.76E-05 | 34.00 |
| SmokingInitiation | rs3847244 | 9:3025368 | European | 0.0187 | 0.0026 | 2.60E-13 | T/C | 0.4700 | 4.34E-05 | 53.50 |
| SmokingInitiation | rs3850736 | 8:64912021 | European | 0.0191 | 0.0026 | 6.43E-14 | G/C | 0.4740 | 4.56E-05 | 56.20 |
| SmokingInitiation | rs3909281 | 5:165096435 | European | 0.0211 | 0.0026 | 1.62E-16 | G/T | 0.5360 | 5.52E-05 | 68.00 |
| SmokingInitiation | rs3934797 | 4:112467612 | European | -0.0213 | 0.0033 | 1.12E-10 | A/G | 0.1820 | 3.38E-05 | 41.60 |
| SmokingInitiation | rs4044321 | 5:166989513 | European | -0.0226 | 0.0027 | 1.75E-17 | G/A | 0.6440 | 5.88E-05 | 72.40 |
| SmokingInitiation | rs4140932 | 4:15458598 | European | -0.0140 | 0.0026 | 4.89E-08 | A/T | 0.4310 | 2.42E-05 | 29.80 |
| SmokingInitiation | rs42417 | 5:94198290 | European | 0.0169 | 0.0028 | 8.27E-10 | T/C | 0.6910 | 3.06E-05 | 37.70 |
| SmokingInitiation | rs4264267 | 13:38359676 | European | 0.0148 | 0.0026 | 6.82E-09 | T/C | 0.5270 | 2.73E-05 | 33.60 |
| SmokingInitiation | rs4310804 | 15:96858409 | European | -0.0182 | 0.0030 | 7.55E-10 | G/C | 0.2470 | 3.08E-05 | 37.90 |
| SmokingInitiation | rs4326350 | 8:10763655 | European | -0.0176 | 0.0026 | 5.16E-12 | G/C | 0.4930 | 3.88E-05 | 47.60 |
| SmokingInitiation | rs4476253 | 18:25253297 | European | -0.0185 | 0.0030 | 5.78E-10 | A/G | 0.2400 | 3.12E-05 | 38.40 |
| SmokingInitiation | rs4543050 | 3:74954560 | European | 0.0222 | 0.0033 | 1.45E-11 | T/A | 0.8160 | 3.70E-05 | 45.60 |
| SmokingInitiation | rs45444697 | 1:155034632 | European | 0.0197 | 0.0031 | 2.72E-10 | G/C | 0.2120 | 3.24E-05 | 39.90 |
| SmokingInitiation | rs4674916 | 2:225365635 | European | -0.0180 | 0.0027 | 3.06E-11 | A/C | 0.3277 | 3.58E-05 | 44.10 |
| SmokingInitiation | rs4674993 | 2:226332033 | European | -0.0240 | 0.0032 | 4.85E-14 | G/A | 0.2000 | 4.61E-05 | 56.80 |
| SmokingInitiation | rs4727189 | 7:88442568 | European | 0.0149 | 0.0027 | 0.00000003 | C/T | 0.3440 | 2.49E-05 | 30.70 |
| SmokingInitiation | rs4752018 | 10:118678712 | European | 0.0189 | 0.0030 | 4.42E-10 | A/C | 0.2310 | 3.16E-05 | 38.90 |
| SmokingInitiation | rs4759229 | 12:56474480 | European | 0.0156 | 0.0027 | 6.53E-09 | G/A | 0.6560 | 2.74E-05 | 33.70 |
| SmokingInitiation | rs4785187 | 16:49766772 | European | 0.0200 | 0.0031 | 6.55E-11 | A/G | 0.2230 | 3.46E-05 | 42.60 |
| SmokingInitiation | rs4788676 | 16:72950468 | European | -0.0177 | 0.0030 | 4.92E-09 | C/T | 0.2285 | 2.78E-05 | 34.20 |
| SmokingInitiation | rs4790874 | 17:1995177 | European | 0.0174 | 0.0026 | 8.43E-12 | T/C | 0.5320 | 3.79E-05 | 46.70 |
| SmokingInitiation | rs4818005 | 21:40588819 | European | -0.0204 | 0.0026 | 1.09E-14 | A/G | 0.5810 | 5.08E-05 | 59.70 |
| SmokingInitiation | rs4822102 | 22:42698430 | European | -0.0165 | 0.0026 | 2.78E-10 | T/C | 0.6180 | 3.23E-05 | 39.80 |
| SmokingInitiation | rs4837631 | 9:122061948 | European | -0.0154 | 0.0026 | 2.03E-09 | T/C | 0.4460 | 2.91E-05 | 35.90 |
| SmokingInitiation | rs4877285 | 9:81354129 | European | -0.0181 | 0.0027 | 2.10E-11 | A/G | 0.6682 | 3.64E-05 | 44.90 |
| SmokingInitiation | rs4886207 | 13:60705792 | European | -0.0162 | 0.0026 | 8.78E-10 | C/T | 0.6370 | 3.05E-05 | 37.60 |
| SmokingInitiation | rs4912332 | 1:58815243 | European | 0.0141 | 0.0025 | 2.94E-08 | T/C | 0.4910 | 2.49E-05 | 30.70 |
| SmokingInitiation | rs540860 | 11:121530888 | European | 0.0176 | 0.0026 | 5.75E-12 | G/A | 0.5430 | 3.85E-05 | 47.40 |
| SmokingInitiation | rs55786907 | 13:59871584 | European | 0.0194 | 0.0035 | 1.84E-08 | G/A | 0.1625 | 2.57E-05 | 31.70 |
| SmokingInitiation | rs55913542 | 14:99693843 | European | 0.0186 | 0.0034 | 3.25E-08 | T/G | 0.1750 | 2.49E-05 | 30.60 |
| SmokingInitiation | rs55944129 | 4:29082156 | European | -0.0176 | 0.0029 | 1.06E-09 | C/T | 0.2670 | 3.02E-05 | 37.20 |
| SmokingInitiation | rs56208390 | 2:83247997 | European | 0.0216 | 0.0039 | 2.68E-08 | G/A | 0.1230 | 2.51E-05 | 30.90 |
| SmokingInitiation | rs56902655 | 15:63898709 | European | -0.0219 | 0.0037 | 4.09E-09 | G/T | 0.1360 | 2.81E-05 | 34.60 |
| SmokingInitiation | rs58400863 | 4:31184484 | European | -0.0202 | 0.0027 | 4.89E-14 | A/G | 0.3470 | 4.61E-05 | 56.80 |
| SmokingInitiation | rs586699 | 11:92289734 | European | -0.0148 | 0.0026 | 7.29E-09 | A/G | 0.5430 | 2.72E-05 | 33.50 |
| SmokingInitiation | rs59537158 | 4:28246049 | European | 0.0225 | 0.0031 | 4.62E-13 | T/C | 0.2140 | 4.25E-05 | 52.40 |
| SmokingInitiation | rs6011779 | 20:61984317 | European | -0.0192 | 0.0032 | 2.83E-09 | T/C | 0.8060 | 2.87E-05 | 35.30 |
| SmokingInitiation | rs6050446 | 20:25195509 | European | 0.0544 | 0.0076 | 8.80E-13 | G/A | 0.9710 | 4.17E-05 | 51.10 |
| SmokingInitiation | rs6073075 | 20:42015801 | European | -0.0187 | 0.0034 | 2.44E-08 | A/T | 0.8240 | 2.54E-05 | 31.10 |
| SmokingInitiation | rs60833441 | 15:74048768 | European | -0.0143 | 0.0026 | 2.28E-08 | G/A | 0.4610 | 2.53E-05 | 31.20 |
| SmokingInitiation | rs61533748 | 2:22582968 | European | 0.0174 | 0.0026 | 2.82E-11 | C/T | 0.3840 | 3.60E-05 | 44.30 |
| SmokingInitiation | rs61884449 | 11:64485193 | European | 0.0200 | 0.0036 | 2.32E-08 | T/C | 0.1492 | 2.53E-05 | 31.20 |
| SmokingInitiation | rs61886926 | 11:64133552 | European | -0.0179 | 0.0026 | 7.30E-12 | T/C | 0.3840 | 3.81E-05 | 46.90 |
| SmokingInitiation | rs619087 | 6:94175279 | European | 0.0143 | 0.0026 | 0.000000031 | G/A | 0.4220 | 2.48E-05 | 30.60 |
| SmokingInitiation | rs61959481 | 13:55834929 | European | -0.0203 | 0.0031 | 7.95E-11 | A/G | 0.2100 | 3.43E-05 | 42.30 |
| SmokingInitiation | rs62007780 | 15:78025464 | European | -0.0159 | 0.0026 | 7.48E-10 | T/G | 0.4160 | 3.08E-05 | 37.90 |
| SmokingInitiation | rs62052916 | 16:72574550 | European | -0.0319 | 0.0050 | 1.62E-10 | T/A | 0.0701 | 3.32E-05 | 40.90 |
| SmokingInitiation | rs62098013 | 18:50863861 | European | 0.0177 | 0.0026 | 2.24E-11 | A/G | 0.3653 | 3.64E-05 | 44.80 |
| SmokingInitiation | rs62106258 | 2:417167 | European | -0.0455 | 0.0060 | 3.33E-14 | C/T | 0.0473 | 4.67E-05 | 57.50 |
| SmokingInitiation | rs62137126 | 2:44250149 | European | -0.0237 | 0.0039 | 1.31E-09 | G/A | 0.1211 | 2.99E-05 | 36.80 |
| SmokingInitiation | rs62180324 | 2:63416606 | European | -0.0195 | 0.0031 | 3.91E-10 | A/G | 0.2120 | 3.18E-05 | 39.20 |
| SmokingInitiation | rs62193862 | 2:202843875 | European | 0.0238 | 0.0042 | 1.99E-08 | A/G | 0.0999 | 2.56E-05 | 31.50 |
| SmokingInitiation | rs62246017 | 3:71483084 | European | -0.0162 | 0.0027 | 3.03E-09 | A/G | 0.3226 | 2.86E-05 | 35.20 |
| SmokingInitiation | rs62340589 | 4:176875795 | European | 0.0174 | 0.0032 | 4.31E-08 | C/G | 0.2010 | 2.43E-05 | 30.00 |
| SmokingInitiation | rs62618693 | 11:32956492 | European | -0.0353 | 0.0063 | 2.09E-08 | T/C | 0.0428 | 2.55E-05 | 31.40 |
| SmokingInitiation | rs6265 | 11:27679916 | European | -0.0293 | 0.0033 | 2.81E-19 | T/C | 0.1880 | 6.54E-05 | 80.60 |
| SmokingInitiation | rs6437769 | 3:107997514 | European | 0.0142 | 0.0026 | 3.74E-08 | T/C | 0.5810 | 2.46E-05 | 30.30 |
| SmokingInitiation | rs6438436 | 3:117822149 | European | 0.0247 | 0.0033 | 5.33E-14 | T/C | 0.8160 | 4.59E-05 | 56.60 |
| SmokingInitiation | rs644740 | 11:65561468 | European | -0.0141 | 0.0026 | 3.67E-08 | T/C | 0.4570 | 2.46E-05 | 30.30 |
| SmokingInitiation | rs6452785 | 5:87685500 | European | -0.0269 | 0.0026 | 4.69E-26 | T/C | 0.4740 | 9.01E-05 | 111.00 |
| SmokingInitiation | rs6497840 | 16:25351633 | European | 0.0228 | 0.0029 | 2.01E-15 | A/G | 0.7070 | 5.37E-05 | 63.10 |
| SmokingInitiation | rs6568832 | 6:97702876 | European | 0.0189 | 0.0030 | 1.74E-10 | A/G | 0.7539 | 3.30E-05 | 40.70 |
| SmokingInitiation | rs67050670 | 18:39297254 | European | -0.0203 | 0.0030 | 2.34E-11 | G/A | 0.2290 | 3.63E-05 | 44.70 |
| SmokingInitiation | rs6731872 | 2:624205 | European | 0.0316 | 0.0034 | 5.35E-21 | G/T | 0.8260 | 7.17E-05 | 88.40 |
| SmokingInitiation | rs6750107 | 2:80748807 | European | 0.0146 | 0.0026 | 0.000000026 | A/G | 0.3869 | 2.52E-05 | 31.00 |
| SmokingInitiation | rs6750529 | 2:182027603 | European | 0.0199 | 0.0029 | 9.26E-12 | T/C | 0.7440 | 3.77E-05 | 46.50 |
| SmokingInitiation | rs6756212 | 2:146140132 | European | -0.0339 | 0.0026 | 3.49E-40 | T/C | 0.5350 | 1.43E-04 | 176.00 |
| SmokingInitiation | rs67777803 | 17:27323322 | European | -0.0246 | 0.0034 | 3.18E-13 | T/G | 0.1720 | 4.31E-05 | 53.10 |
| SmokingInitiation | rs6782116 | 3:77176032 | European | -0.0147 | 0.0026 | 1.46E-08 | T/C | 0.4150 | 2.61E-05 | 32.10 |
| SmokingInitiation | rs6874731 | 5:80263865 | European | 0.0153 | 0.0025 | 1.83E-09 | G/T | 0.4840 | 2.93E-05 | 36.10 |
| SmokingInitiation | rs6890961 | 5:166778503 | European | -0.0193 | 0.0026 | 2.13E-13 | T/C | 0.6240 | 4.37E-05 | 53.90 |
| SmokingInitiation | rs6936160 | 6:100347745 | European | 0.0201 | 0.0028 | 4.20E-13 | T/C | 0.6980 | 4.26E-05 | 52.50 |
| SmokingInitiation | rs6948707 | 7:1870794 | European | 0.0243 | 0.0026 | 4.24E-21 | G/T | 0.4190 | 7.21E-05 | 88.90 |
| SmokingInitiation | rs6968380 | 7:114940159 | European | -0.0234 | 0.0027 | 1.05E-17 | A/G | 0.6810 | 5.96E-05 | 73.40 |
| SmokingInitiation | rs6986430 | 8:93048104 | European | -0.0243 | 0.0031 | 1.99E-15 | C/T | 0.2224 | 5.12E-05 | 63.10 |
| SmokingInitiation | rs7024924 | 9:8282399 | European | 0.0189 | 0.0034 | 0.000000019 | C/T | 0.1740 | 2.56E-05 | 31.60 |
| SmokingInitiation | rs7026534 | 9:134907263 | European | -0.0166 | 0.0028 | 2.68E-09 | G/T | 0.7038 | 2.87E-05 | 35.40 |
| SmokingInitiation | rs7072776 | 10:22032942 | European | -0.0220 | 0.0028 | 5.66E-15 | G/A | 0.7120 | 4.95E-05 | 61.00 |
| SmokingInitiation | rs7134009 | 12:75263193 | European | -0.0158 | 0.0029 | 0.000000043 | C/T | 0.2870 | 2.55E-05 | 30.00 |
| SmokingInitiation | rs71367544 | 18:77574374 | European | 0.0206 | 0.0032 | 8.54E-11 | T/C | 0.2030 | 3.42E-05 | 42.10 |
| SmokingInitiation | rs71592686 | 5:60121271 | European | 0.0207 | 0.0029 | 3.85E-13 | C/T | 0.2740 | 4.28E-05 | 52.70 |
| SmokingInitiation | rs71602617 | 4:136406155 | European | -0.0178 | 0.0032 | 0.000000021 | T/C | 0.2160 | 2.67E-05 | 31.40 |
| SmokingInitiation | rs7188873 | 16:24727064 | European | 0.0203 | 0.0026 | 8.46E-15 | G/A | 0.6130 | 4.89E-05 | 60.20 |
| SmokingInitiation | rs7192140 | 16:10173748 | European | -0.0169 | 0.0025 | 3.40E-11 | C/T | 0.4980 | 3.56E-05 | 43.90 |
| SmokingInitiation | rs72780746 | 5:103929588 | European | -0.0258 | 0.0034 | 2.05E-14 | C/T | 0.1730 | 4.75E-05 | 58.50 |
| SmokingInitiation | rs72789626 | 5:106825618 | European | -0.0256 | 0.0037 | 5.13E-12 | A/T | 0.1360 | 3.86E-05 | 47.60 |
| SmokingInitiation | rs72790288 | 2:29513404 | European | -0.0455 | 0.0077 | 3.28E-09 | A/G | 0.0282 | 2.84E-05 | 35.00 |
| SmokingInitiation | rs72898831 | 18:42658643 | European | -0.0244 | 0.0035 | 4.14E-12 | G/A | 0.1550 | 3.90E-05 | 48.10 |
| SmokingInitiation | rs72938304 | 18:53661743 | European | -0.0272 | 0.0040 | 1.36E-11 | A/G | 0.1130 | 3.71E-05 | 45.70 |
| SmokingInitiation | rs73008357 | 6:156431856 | European | -0.0223 | 0.0040 | 2.44E-08 | C/A | 0.1210 | 2.65E-05 | 31.10 |
| SmokingInitiation | rs7333559 | 13:100546450 | European | -0.0232 | 0.0031 | 5.94E-14 | A/G | 0.7830 | 4.58E-05 | 56.40 |
| SmokingInitiation | rs73831818 | 3:55988394 | European | 0.0320 | 0.0055 | 5.46E-09 | G/A | 0.0570 | 2.76E-05 | 34.00 |
| SmokingInitiation | rs748832 | 3:16851202 | European | 0.0172 | 0.0026 | 6.60E-11 | G/A | 0.3710 | 3.46E-05 | 42.60 |
| SmokingInitiation | rs7505855 | 18:31696075 | European | -0.0170 | 0.0026 | 5.31E-11 | T/C | 0.5860 | 3.50E-05 | 43.10 |
| SmokingInitiation | rs75674569 | 13:96823724 | European | -0.0253 | 0.0043 | 2.58E-09 | A/G | 0.0997 | 2.88E-05 | 35.50 |
| SmokingInitiation | rs75919030 | 17:50193197 | European | -0.0210 | 0.0029 | 3.35E-13 | C/T | 0.2670 | 4.30E-05 | 53.00 |
| SmokingInitiation | rs7598402 | 2:50735943 | European | -0.0147 | 0.0025 | 7.38E-09 | G/C | 0.4921 | 2.71E-05 | 33.40 |
| SmokingInitiation | rs7600835 | 2:172521827 | European | -0.0151 | 0.0027 | 0.000000018 | A/G | 0.3420 | 2.57E-05 | 31.70 |
| SmokingInitiation | rs7631379 | 3:181409057 | European | 0.0208 | 0.0032 | 3.94E-11 | C/T | 0.2060 | 3.54E-05 | 43.60 |
| SmokingInitiation | rs7640107 | 3:59966156 | European | -0.0142 | 0.0026 | 3.46E-08 | T/C | 0.4308 | 2.47E-05 | 30.40 |
| SmokingInitiation | rs76460663 | 11:111979741 | European | -0.0423 | 0.0064 | 4.15E-11 | G/C | 0.0411 | 3.53E-05 | 43.50 |
| SmokingInitiation | rs7657022 | 4:35501032 | European | 0.0183 | 0.0025 | 7.34E-13 | G/A | 0.4890 | 4.18E-05 | 51.50 |
| SmokingInitiation | rs76608582 | 19:4474725 | European | -0.0345 | 0.0059 | 4.88E-09 | A/C | 0.0489 | 2.78E-05 | 34.20 |
| SmokingInitiation | rs76841737 | 7:91281409 | European | -0.0231 | 0.0042 | 3.26E-08 | G/C | 0.1030 | 2.48E-05 | 30.50 |
| SmokingInitiation | rs7696257 | 4:137474783 | European | 0.0153 | 0.0026 | 6.78E-09 | A/G | 0.3660 | 2.73E-05 | 33.60 |
| SmokingInitiation | rs77215829 | 12:112618346 | European | -0.0240 | 0.0038 | 2.02E-10 | C/A | 0.1310 | 3.29E-05 | 40.40 |
| SmokingInitiation | rs77283305 | 7:132593831 | European | -0.0152 | 0.0028 | 3.91E-08 | A/G | 0.3058 | 2.45E-05 | 30.20 |
| SmokingInitiation | rs7743165 | 6:67521222 | European | 0.0193 | 0.0025 | 4.15E-14 | G/T | 0.4950 | 4.63E-05 | 57.10 |
| SmokingInitiation | rs7802996 | 7:77771983 | European | -0.0209 | 0.0034 | 1.06E-09 | T/C | 0.1660 | 3.02E-05 | 37.20 |
| SmokingInitiation | rs7809303 | 7:69484366 | European | -0.0214 | 0.0027 | 3.48E-15 | A/G | 0.3250 | 5.03E-05 | 62.00 |
| SmokingInitiation | rs78239456 | 11:112984491 | European | -0.0185 | 0.0027 | 9.37E-12 | T/A | 0.3765 | 4.02E-05 | 46.50 |
| SmokingInitiation | rs7867822 | 9:20676454 | European | -0.0151 | 0.0027 | 2.76E-08 | G/A | 0.6730 | 2.51E-05 | 30.90 |
| SmokingInitiation | rs7920501 | 10:10043159 | European | -0.0155 | 0.0026 | 1.25E-09 | A/T | 0.4650 | 2.99E-05 | 36.90 |
| SmokingInitiation | rs7921378 | 10:63674885 | European | -0.0233 | 0.0025 | 6.10E-20 | C/G | 0.4820 | 6.78E-05 | 83.60 |
| SmokingInitiation | rs7929518 | 11:85980958 | European | 0.0192 | 0.0030 | 2.55E-10 | G/A | 0.7730 | 3.25E-05 | 40.00 |
| SmokingInitiation | rs7943721 | 11:73309393 | European | -0.0212 | 0.0034 | 3.58E-10 | A/G | 0.8290 | 3.19E-05 | 39.30 |
| SmokingInitiation | rs7969559 | 12:69655167 | European | -0.0170 | 0.0028 | 1.53E-09 | G/A | 0.7130 | 2.96E-05 | 36.50 |
| SmokingInitiation | rs8005334 | 14:79563654 | European | 0.0167 | 0.0027 | 3.44E-10 | G/T | 0.3600 | 3.20E-05 | 39.40 |
| SmokingInitiation | rs8027457 | 15:99204101 | European | 0.0153 | 0.0025 | 1.88E-09 | C/T | 0.5110 | 2.93E-05 | 36.10 |
| SmokingInitiation | rs8050598 | 16:49891964 | European | 0.0187 | 0.0029 | 1.76E-10 | T/C | 0.2541 | 3.30E-05 | 40.70 |
| SmokingInitiation | rs8096225 | 18:36921851 | European | 0.0155 | 0.0028 | 2.63E-08 | C/A | 0.7030 | 2.52E-05 | 31.00 |
| SmokingInitiation | rs8103660 | 19:18566395 | European | 0.0158 | 0.0027 | 3.03E-09 | C/T | 0.3544 | 2.86E-05 | 35.20 |
| SmokingInitiation | rs876793 | 1:237852083 | European | -0.0179 | 0.0027 | 5.69E-11 | C/T | 0.3493 | 3.65E-05 | 42.90 |
| SmokingInitiation | rs910912 | 20:54462393 | European | -0.0168 | 0.0029 | 7.82E-09 | C/T | 0.7390 | 2.71E-05 | 33.30 |
| SmokingInitiation | rs925524 | 1:46496709 | European | 0.0156 | 0.0028 | 2.94E-08 | G/A | 0.7100 | 2.49E-05 | 30.70 |
| SmokingInitiation | rs9288999 | 3:114147927 | European | 0.0174 | 0.0029 | 1.50E-09 | A/G | 0.7350 | 2.96E-05 | 36.50 |
| SmokingInitiation | rs9302604 | 16:69576894 | European | 0.0187 | 0.0026 | 3.29E-13 | G/A | 0.4350 | 4.30E-05 | 53.00 |
| SmokingInitiation | rs9323328 | 14:58653514 | European | -0.0142 | 0.0026 | 2.55E-08 | G/A | 0.5370 | 2.52E-05 | 31.00 |
| SmokingInitiation | rs9331343 | 6:157738258 | European | -0.0141 | 0.0026 | 0.000000039 | C/T | 0.5680 | 2.45E-05 | 30.20 |
| SmokingInitiation | rs9423279 | 10:125680419 | European | -0.0186 | 0.0027 | 3.06E-12 | G/C | 0.6450 | 3.95E-05 | 48.70 |
| SmokingInitiation | rs951740 | 1:44011737 | European | 0.0295 | 0.0026 | 3.82E-29 | A/G | 0.6250 | 1.02E-04 | 126.00 |
| SmokingInitiation | rs9538162 | 13:59265043 | European | 0.0174 | 0.0026 | 1.76E-11 | C/T | 0.4159 | 3.67E-05 | 45.20 |
| SmokingInitiation | rs9540731 | 13:66949370 | European | -0.0177 | 0.0025 | 3.42E-12 | T/C | 0.5090 | 3.93E-05 | 48.40 |
| SmokingInitiation | rs9545155 | 13:80191873 | European | -0.0161 | 0.0026 | 3.04E-10 | C/T | 0.4780 | 3.22E-05 | 39.70 |
| SmokingInitiation | rs9627272 | 22:46442288 | European | -0.0155 | 0.0026 | 2.42E-09 | C/G | 0.4070 | 2.89E-05 | 35.60 |
| SmokingInitiation | rs9826984 | 3:131945722 | European | -0.0141 | 0.0026 | 3.87E-08 | A/G | 0.5420 | 2.45E-05 | 30.20 |
| SmokingInitiation | rs9841807 | 3:175718927 | European | 0.0163 | 0.0029 | 1.35E-08 | T/C | 0.2730 | 2.62E-05 | 32.30 |
| SmokingInitiation | rs9850597 | 3:161761866 | European | -0.0186 | 0.0033 | 1.65E-08 | A/G | 0.8160 | 2.59E-05 | 31.90 |
| SmokingInitiation | rs986714 | 5:50821338 | European | -0.0160 | 0.0026 | 4.13E-10 | T/A | 0.4450 | 3.17E-05 | 39.10 |
| SmokingInitiation | rs9922607 | 16:17570220 | European | -0.0222 | 0.0032 | 3.42E-12 | T/C | 0.2000 | 3.93E-05 | 48.40 |
| SmokingInitiation | rs9941217 | 16:18050926 | European | -0.0186 | 0.0027 | 3.50E-12 | G/C | 0.3522 | 3.93E-05 | 48.40 |
| SmokingInitiation | rs9987376 | 8:93190014 | European | -0.0205 | 0.0026 | 2.01E-15 | G/T | 0.5743 | 5.12E-05 | 63.10 |
| DrinksPerWeek | rs10004020 | 4:152968372 | European | 0.0091 | 0.0016 | 2.43E-08 | A/G | 0.7200 | 3.30E-05 | 31.10 |
| DrinksPerWeek | rs10506274 | 12:81601464 | European | -0.0090 | 0.0015 | 5.78E-10 | T/G | 0.4840 | 4.08E-05 | 38.40 |
| DrinksPerWeek | rs10750025 | 11:113424042 | European | 0.0103 | 0.0016 | 4.89E-11 | T/C | 0.6860 | 4.59E-05 | 43.20 |
| DrinksPerWeek | rs10753661 | 1:165119792 | European | -0.0086 | 0.0016 | 3.76E-08 | A/G | 0.6840 | 3.23E-05 | 30.30 |
| DrinksPerWeek | rs10876188 | 12:51895882 | European | -0.0080 | 0.0015 | 4.84E-08 | T/C | 0.4570 | 3.17E-05 | 29.80 |
| DrinksPerWeek | rs10978550 | 9:109345993 | European | -0.0117 | 0.0018 | 7.15E-11 | C/T | 0.2060 | 4.51E-05 | 42.50 |
| DrinksPerWeek | rs11030084 | 11:27643725 | European | -0.0106 | 0.0019 | 1.72E-08 | T/C | 0.1840 | 3.38E-05 | 31.80 |
| DrinksPerWeek | rs1104608 | 16:73912588 | European | -0.0110 | 0.0015 | 1.05E-13 | C/G | 0.4250 | 5.89E-05 | 55.30 |
| DrinksPerWeek | rs1123285 | 14:57274519 | European | -0.0089 | 0.0015 | 8.14E-09 | G/C | 0.3350 | 3.53E-05 | 33.20 |
| DrinksPerWeek | rs113443718 | 16:29892184 | European | -0.0102 | 0.0016 | 1.19E-10 | A/G | 0.3050 | 4.42E-05 | 41.50 |
| DrinksPerWeek | rs11625650 | 14:104610138 | European | -0.0096 | 0.0017 | 2.89E-08 | A/G | 0.2330 | 3.27E-05 | 30.80 |
| DrinksPerWeek | rs11692435 | 2:98275354 | European | 0.0174 | 0.0026 | 2.53E-11 | A/G | 0.0852 | 4.75E-05 | 44.50 |
| DrinksPerWeek | rs11940694 | 4:39414993 | European | 0.0259 | 0.0015 | 3.03E-68 | G/A | 0.5970 | 3.24E-04 | 305.00 |
| DrinksPerWeek | rs12088813 | 1:66407700 | European | -0.0093 | 0.0016 | 1.58E-08 | C/A | 0.2670 | 3.41E-05 | 32.00 |
| DrinksPerWeek | rs1217091 | 8:64527399 | European | 0.0122 | 0.0019 | 7.05E-11 | C/T | 0.8120 | 4.51E-05 | 42.50 |
| DrinksPerWeek | rs1229984 | 4:100239319 | European | 0.1505 | 0.0039 | <2.2e-308 | C/T | 0.9630 | 1.61E-03 | 1520.00 |
| DrinksPerWeek | rs1260326 | 2:27730940 | European | 0.0209 | 0.0015 | 8.05E-45 | C/T | 0.6010 | 2.09E-04 | 197.00 |
| DrinksPerWeek | rs12651313 | 4:171086393 | European | -0.0086 | 0.0015 | 3.79E-09 | G/C | 0.4430 | 3.69E-05 | 34.70 |
| DrinksPerWeek | rs12655091 | 5:144412335 | European | -0.0083 | 0.0015 | 1.25E-08 | A/G | 0.5300 | 3.44E-05 | 32.40 |
| DrinksPerWeek | rs12795042 | 11:133658168 | European | -0.0083 | 0.0015 | 3.25E-08 | C/A | 0.6230 | 3.25E-05 | 30.60 |
| DrinksPerWeek | rs12907323 | 15:86796012 | European | 0.0085 | 0.0015 | 9.93E-09 | G/A | 0.4110 | 3.50E-05 | 32.90 |
| DrinksPerWeek | rs13024996 | 2:144225215 | European | -0.0109 | 0.0015 | 5.72E-13 | A/C | 0.3640 | 5.51E-05 | 51.90 |
| DrinksPerWeek | rs13032049 | 2:63581507 | European | 0.0102 | 0.0016 | 3.00E-10 | G/A | 0.2830 | 4.22E-05 | 39.70 |
| DrinksPerWeek | rs13066454 | 3:93994255 | European | -0.0088 | 0.0015 | 4.13E-09 | T/C | 0.3980 | 3.69E-05 | 34.60 |
| DrinksPerWeek | rs13094887 | 3:70968431 | European | -0.0103 | 0.0016 | 8.57E-11 | T/A | 0.3010 | 4.47E-05 | 42.10 |
| DrinksPerWeek | rs13107325 | 4:103188709 | European | -0.0275 | 0.0028 | 1.53E-22 | T/C | 0.0722 | 1.01E-04 | 95.40 |
| DrinksPerWeek | rs13250583 | 8:20949917 | European | -0.0097 | 0.0018 | 0.000000047 | T/C | 0.2130 | 3.17E-05 | 29.80 |
| DrinksPerWeek | rs13383034 | 2:45155276 | European | 0.0149 | 0.0016 | 6.31E-22 | T/C | 0.3290 | 9.84E-05 | 92.60 |
| DrinksPerWeek | rs1713676 | 11:113660576 | European | -0.0080 | 0.0015 | 4.29E-08 | G/A | 0.5225 | 3.19E-05 | 30.00 |
| DrinksPerWeek | rs17177078 | 16:24810681 | European | -0.0223 | 0.0030 | 1.27E-13 | T/C | 0.0626 | 5.84E-05 | 54.90 |
| DrinksPerWeek | rs17665139 | 10:125093880 | European | -0.0116 | 0.0020 | 1.59E-08 | T/C | 0.1490 | 3.39E-05 | 31.90 |
| DrinksPerWeek | rs2011092 | 3:141124607 | European | -0.0089 | 0.0015 | 7.35E-09 | C/T | 0.3386 | 3.55E-05 | 33.40 |
| DrinksPerWeek | rs2165670 | 4:100286085 | European | 0.0231 | 0.0024 | 1.67E-22 | A/G | 0.1063 | 1.01E-04 | 95.30 |
| DrinksPerWeek | rs2180870 | 14:58782779 | European | -0.0122 | 0.0021 | 1.12E-08 | C/T | 0.1350 | 3.46E-05 | 32.60 |
| DrinksPerWeek | rs2472297 | 15:75027880 | European | 0.0106 | 0.0017 | 3.10E-10 | T/C | 0.2490 | 4.21E-05 | 39.60 |
| DrinksPerWeek | rs2532276 | 17:44246624 | European | -0.0218 | 0.0026 | 1.62E-17 | A/C | 0.2150 | 1.60E-04 | 72.60 |
| DrinksPerWeek | rs2764771 | 16:20013793 | European | 0.0099 | 0.0016 | 4.02E-10 | A/G | 0.3070 | 4.16E-05 | 39.10 |
| DrinksPerWeek | rs281379 | 19:49214274 | European | 0.0137 | 0.0015 | 4.91E-21 | A/G | 0.5080 | 9.41E-05 | 88.60 |
| DrinksPerWeek | rs2854334 | 17:29715500 | European | 0.0092 | 0.0015 | 7.51E-10 | G/A | 0.6150 | 4.03E-05 | 37.90 |
| DrinksPerWeek | rs28601761 | 8:126500031 | European | 0.0091 | 0.0015 | 7.17E-10 | G/C | 0.4200 | 4.04E-05 | 38.00 |
| DrinksPerWeek | rs28680958 | 1:173848808 | European | -0.0110 | 0.0018 | 5.13E-10 | A/G | 0.2170 | 4.11E-05 | 38.60 |
| DrinksPerWeek | rs28929474 | 14:94844947 | European | -0.0368 | 0.0054 | 1.34E-11 | T/C | 0.0183 | 4.87E-05 | 45.80 |
| DrinksPerWeek | rs35034355 | 7:103840115 | European | -0.0081 | 0.0015 | 2.87E-08 | A/G | 0.5210 | 3.27E-05 | 30.80 |
| DrinksPerWeek | rs36052336 | 4:100273594 | European | -0.0184 | 0.0030 | 1.23E-09 | G/A | 0.0615 | 3.92E-05 | 36.90 |
| DrinksPerWeek | rs3748034 | 4:3446091 | European | -0.0117 | 0.0021 | 1.67E-08 | T/G | 0.1430 | 3.38E-05 | 31.80 |
| DrinksPerWeek | rs378421 | 16:28754684 | European | -0.0112 | 0.0015 | 4.83E-14 | A/G | 0.4040 | 6.05E-05 | 56.80 |
| DrinksPerWeek | rs3803800 | 17:7462969 | European | 0.0114 | 0.0018 | 1.50E-10 | G/A | 0.7860 | 4.36E-05 | 41.00 |
| DrinksPerWeek | rs3809162 | 12:54674235 | European | 0.0091 | 0.0015 | 1.19E-09 | G/A | 0.3970 | 3.93E-05 | 37.00 |
| DrinksPerWeek | rs4092465 | 18:55080437 | European | -0.0083 | 0.0015 | 4.39E-08 | G/A | 0.6350 | 3.19E-05 | 30.00 |
| DrinksPerWeek | rs4501255 | 4:42151306 | European | 0.0107 | 0.0017 | 4.83E-10 | G/C | 0.2350 | 4.11E-05 | 38.70 |
| DrinksPerWeek | rs4548913 | 17:2209888 | European | -0.0084 | 0.0015 | 3.11E-08 | A/G | 0.6320 | 3.25E-05 | 30.60 |
| DrinksPerWeek | rs4690727 | 4:143648579 | European | 0.0108 | 0.0016 | 2.43E-11 | G/C | 0.7180 | 4.74E-05 | 44.60 |
| DrinksPerWeek | rs4699791 | 4:101243023 | European | 0.0186 | 0.0025 | 6.58E-14 | A/G | 0.0957 | 5.97E-05 | 56.20 |
| DrinksPerWeek | rs4815364 | 20:25035711 | European | 0.0086 | 0.0015 | 1.02E-08 | A/G | 0.6160 | 3.48E-05 | 32.80 |
| DrinksPerWeek | rs4842786 | 12:92170791 | European | -0.0088 | 0.0015 | 2.73E-09 | A/G | 0.5840 | 3.76E-05 | 35.40 |
| DrinksPerWeek | rs4916723 | 5:87854395 | European | -0.0100 | 0.0015 | 1.72E-11 | C/A | 0.4160 | 4.81E-05 | 45.30 |
| DrinksPerWeek | rs4938230 | 11:116075001 | European | 0.0128 | 0.0020 | 1.48E-10 | A/C | 0.8420 | 4.37E-05 | 41.10 |
| DrinksPerWeek | rs500321 | 13:27124360 | European | -0.0097 | 0.0017 | 4.92E-09 | T/A | 0.7360 | 3.63E-05 | 34.20 |
| DrinksPerWeek | rs5024204 | 1:71491890 | European | 0.0097 | 0.0016 | 2.55E-09 | T/A | 0.2780 | 3.78E-05 | 35.50 |
| DrinksPerWeek | rs55872084 | 5:155902003 | European | 0.0100 | 0.0017 | 6.32E-09 | T/G | 0.2350 | 3.58E-05 | 33.70 |
| DrinksPerWeek | rs55932213 | 9:108755622 | European | 0.0095 | 0.0017 | 9.55E-09 | G/A | 0.7364 | 3.50E-05 | 32.90 |
| DrinksPerWeek | rs56030824 | 11:47397353 | European | -0.0116 | 0.0016 | 1.15E-13 | A/G | 0.3220 | 5.88E-05 | 55.10 |
| DrinksPerWeek | rs56337305 | 2:225475560 | European | -0.0096 | 0.0015 | 1.63E-10 | C/T | 0.3830 | 4.34E-05 | 40.90 |
| DrinksPerWeek | rs58107686 | 1:33837334 | European | -0.0097 | 0.0016 | 7.79E-10 | A/C | 0.3280 | 4.19E-05 | 37.80 |
| DrinksPerWeek | rs62044525 | 16:64872590 | European | -0.0122 | 0.0019 | 1.03E-10 | G/C | 0.1840 | 4.45E-05 | 41.80 |
| DrinksPerWeek | rs62250685 | 3:85457240 | European | -0.0144 | 0.0015 | 1.05E-21 | G/A | 0.6140 | 9.77E-05 | 91.60 |
| DrinksPerWeek | rs6460047 | 7:73042443 | European | 0.0116 | 0.0018 | 9.69E-11 | C/T | 0.2080 | 4.45E-05 | 41.90 |
| DrinksPerWeek | rs6787172 | 3:158187811 | European | -0.0080 | 0.0015 | 4.27E-08 | G/T | 0.5540 | 3.19E-05 | 30.00 |
| DrinksPerWeek | rs682011 | 11:121544285 | European | 0.0082 | 0.0015 | 2.22E-08 | C/T | 0.5590 | 3.33E-05 | 31.30 |
| DrinksPerWeek | rs6951574 | 7:153489744 | European | 0.0132 | 0.0015 | 1.58E-19 | C/T | 0.4580 | 8.68E-05 | 81.70 |
| DrinksPerWeek | rs705687 | 1:4548453 | European | -0.0109 | 0.0018 | 8.15E-10 | G/A | 0.7850 | 4.01E-05 | 37.70 |
| DrinksPerWeek | rs7074871 | 10:110507806 | European | -0.0094 | 0.0017 | 1.86E-08 | A/G | 0.2550 | 3.36E-05 | 31.60 |
| DrinksPerWeek | rs7185555 | 16:69131281 | European | -0.0111 | 0.0020 | 4.24E-08 | C/G | 0.1530 | 3.19E-05 | 30.00 |
| DrinksPerWeek | rs72859280 | 2:147956293 | European | 0.0229 | 0.0039 | 4.44E-09 | T/G | 0.0362 | 3.65E-05 | 34.40 |
| DrinksPerWeek | rs77165542 | 2:430975 | European | -0.0260 | 0.0040 | 5.63E-11 | T/C | 0.0349 | 4.56E-05 | 42.90 |
| DrinksPerWeek | rs79139602 | 4:100444363 | European | 0.0603 | 0.0051 | 1.80E-32 | T/A | 0.0211 | 1.50E-04 | 141.00 |
| DrinksPerWeek | rs7950166 | 11:8642218 | European | -0.0098 | 0.0015 | 9.89E-11 | T/C | 0.6370 | 4.44E-05 | 41.80 |
| DrinksPerWeek | rs79616692 | 16:72338507 | European | 0.0163 | 0.0024 | 4.11E-12 | C/G | 0.1080 | 5.12E-05 | 48.10 |
| DrinksPerWeek | rs823114 | 1:205719532 | European | 0.0088 | 0.0015 | 2.31E-09 | A/G | 0.5530 | 3.80E-05 | 35.70 |
| DrinksPerWeek | rs828867 | 2:74334462 | European | 0.0088 | 0.0015 | 2.15E-09 | A/G | 0.5450 | 3.80E-05 | 35.80 |
| DrinksPerWeek | rs9607814 | 22:41946519 | European | -0.0102 | 0.0019 | 4.31E-08 | A/C | 0.2000 | 3.32E-05 | 30.00 |
| DrinksPerWeek | rs9838144 | 3:131576287 | European | -0.0100 | 0.0018 | 2.65E-08 | C/G | 0.2090 | 3.28E-05 | 30.90 |
| DrinksPerWeek | rs9950000 | 18:53052169 | European | -0.0091 | 0.0015 | 9.38E-10 | T/C | 0.3950 | 3.97E-05 | 37.40 |
| CoffeeConsumption | rs4410790 | 7:17284577 | European | 0.1000 | 0.0100 | 1.48E-57 | C/T | 0.6300 | 7.72E-04 | 100.00 |
| CoffeeConsumption | rs7800944 | 7:73035857 | European | 0.0500 | 0.0100 | 7.82E-09 | C/T | 0.2800 | 1.93E-04 | 25.00 |
| CoffeeConsumption | rs17685 | 7:75616105 | European | 0.0700 | 0.0100 | 9.06E-14 | A/G | 0.2900 | 3.78E-04 | 49.00 |
| CoffeeConsumption | rs2472297 | 15:75027880 | European | 0.1400 | 0.0100 | 6.89E-44 | T/C | 0.2400 | 1.51E-03 | 196.00 |

Supplementary Table 2. Summary for directional horizontal pleiotropy tests

| **Outcome** | **Method** | **smoking initiation** | **alcohol drinking** | **coffee consumption** |
| --- | --- | --- | --- | --- |
|  |  | ***P-*pleiotropy** | ***P-*pleiotropy** | ***P-*pleiotropy** |
| SHBG | MR-Egger Intercept | 6.71E-01 | 5.06E-01 | 9.64E-01 |
|  | MR-PRESSO | <0.001 | <0.001 | 3.11E-01 |
| Bio-T | MR-Egger Intercept | 8.94E-01 | 2.90E-01 | 7.88E-02 |
|  | MR-PRESSO | <0.001 | <0.001 | 1.00E-03 |
| TT | MR-Egger Intercept | 3.14E-01 | 8.84E-02 | 2.84E-02 |
|  | MR-PRESSO | <0.001 | <0.001 | <0.001 |
| E_2_ | MR-Egger Intercept | 2.84E-02 | 7.16E-01 | 2.10E-01 |
|  | MR-PRESSO | 2.11E-01 | 7.41E-01 | - |
| AMH | MR-Egger Intercept | 1.69E-01 | 5.77E-01 | 9.76E-01 |
|  | MR-PRESSO | 4.76E-01 | 5.55E-01 | 7.12E-01 |
| Age at menopause | MR-Egger Intercept | 3.48E-02 | 7.79E-01 | 5.85E-01 |
|  | MR-PRESSO | <0.001 | <0.001 | 9.26E-01 |
| Irregular menstrual cycle/bleeding | MR-Egger Intercept | 8.42E-02 | 8.49E-01 | 9.76E-01 |
|  | MR-PRESSO | 6.80E-02 | 4.87E-01 | 9.59E-01 |
| Dysmenorrhea | MR-Egger Intercept | 5.24E-01 | 8.90E-01 | 6.05E-01 |
|  | MR-PRESSO | 7.15E-01 | 8.16E-01 | 5.88E-01 |

Supplementary Table 3. Heterogeneity estimation for each group

| **Outcome** | **Method** | **smoking initiation** | | | **alcohol drinking** | | | **coffee consumption** | | |
| --- | --- | --- | --- | --- | --- | --- | --- | --- | --- | --- |
|  |  | **Q** | **Q_df** | **Q_pval** | **Q** | **Q_df** | **Q_pval** | **Q** | **Q_df** | **Q_pval** |
| SHBG | MR-Egger | 971.135 | 298 | 2.72E-72 | 2833.282 | 78 | 0.00E+00 | 5.245 | 2 | 7.26E-02 |
|  | IVW | 971.726 | 299 | 4.02E-72 | 2849.498 | 79 | 0.00E+00 | 5.252 | 3 | 1.54E-01 |
| Bio-T | MR-Egger | 1044.523 | 298 | 1.47E-83 | 1095.484 | 78 | 3.14E-179 | 5.868 | 2 | 5.32E-02 |
|  | IVW | 1044.586 | 299 | 2.71E-83 | 1111.408 | 79 | 7.17E-182 | 38.772 | 3 | 1.94E-08 |
| TT | MR-Egger | 1625.715 | 298 | 2.20E-181 | 482.993 | 78 | 1.06E-59 | 4.494 | 2 | 1.06E-01 |
|  | IVW | 1631.267 | 299 | 5.31E-182 | 501.434 | 79 | 1.10E-62 | 80.348 | 3 | 2.58E-17 |
| E_2_ | MR-Egger | 261.554 | 247 | 2.51E-01 | 54.824 | 61 | 6.98E-01 | 1.159 | 1 | 2.82E-01 |
|  | IVW | 266.704 | 248 | 1.98E-01 | 54.958 | 62 | 7.25E-01 | 11.056 | 2 | 3.97E-03 |
| AMH | MR-Egger | 296.179 | 298 | 5.19E-01 | 78.417 | 80 | 5.29E-01 | 1.720 | 2 | 4.23E-01 |
|  | IVW | 298.077 | 299 | 5.04E-01 | 78.730 | 81 | 5.51E-01 | 1.721 | 3 | 6.32E-01 |
| Age at menopause | MR-Egger | 545.234 | 298 | 9.87E-17 | 162.771 | 78 | 6.34E-08 | 0.189 | 2 | 9.10E-01 |
|  | IVW | 553.457 | 299 | 1.99E-17 | 162.936 | 79 | 8.89E-08 | 0.604 | 3 | 8.96E-01 |
| Irregular menstrual cycle/bleeding | MR-Egger | 330.453 | 295 | 7.61E-02 | 80.574 | 79 | 4.30E-01 | 0.190 | 2 | 9.10E-01 |
|  | IVW | 333.816 | 296 | 6.43E-02 | 80.611 | 80 | 4.60E-01 | 0.191 | 3 | 9.79E-01 |
| Dysmenorrhea | MR-Egger | 281.722 | 295 | 7.01E-01 | 69.218 | 79 | 7.76E-01 | 2.061 | 2 | 3.57E-01 |
|  | IVW | 282.130 | 296 | 7.09E-01 | 69.237 | 80 | 7.99E-01 | 2.442 | 3 | 4.86E-01 |

**Supplementary Figure Legends**

**Supplementary Figure 1. Leave-one-out analysis plot.** Association between SNPs associated with smoking initiation and risk of (A) SHBG, (B) bio-T, (C) TT, (D) E_2_, (E) AMH, (F) age at menopause, (G) irregular menstrual cycle/bleeding and (H) dysmenorrhea.

**Supplementary Figure 2. Leave-one-out analysis plot.** Association between SNPs associated with alcohol drinking and risk of (A) SHBG, (B) bio-T, (C) TT, (D) E_2_, (E) AMH, (F) age at menopause, (G) irregular menstrual cycle/bleeding and (H) dysmenorrhea.

**Supplementary Figure 3. Leave-one-out analysis plot.** Association between SNPs associated with coffee consumption and risk of (A) SHBG, (B) bio-T, (C) TT, (D) E_2_, (E) AMH, (F) age at menopause, (G) irregular menstrual cycle/bleeding and (H) dysmenorrhea.

**Supplementary Figure 4. Scatter plot.** Association between SNPs associated with smoking initiation and risk of (A) SHBG, (B) bio-T, (C) TT, (D) E_2_, (E) AMH, (F) age at menopause, (G) irregular menstrual cycle/bleeding and (H) dysmenorrhea.

**Supplementary Figure 5. Scatter plot.** Association between SNPs associated with alcohol drinking and risk of (A) SHBG, (B) bio-T, (C) TT, (D) E_2_, (E) AMH, (F) age at menopause, (G) irregular menstrual cycle/bleeding and (H) dysmenorrhea.

**Supplementary Figure 6. Scatter plot.** Association between SNPs associated with coffee consumption and risk of (A) SHBG, (B) bio-T, (C) TT, (D) E_2_, (E) AMH, (F) age at menopause, (G) irregular menstrual cycle/bleeding and (H) dysmenorrhea.

**Supplementary Figure 7. Forest plot.** Association between SNPs associated with smoking initiation and risk of (A) SHBG, (B) bio-T, (C) TT, (D) E_2_, (E) AMH, (F) age at menopause, (G) irregular menstrual cycle/bleeding and (H) dysmenorrhea.

**Supplementary Figure 8. Forest plot.** Association between SNPs associated with alcohol drinking and risk of (A) SHBG, (B) bio-T, (C) TT, (D) E_2_, (E) AMH, (F) age at menopause, (G) irregular menstrual cycle/bleeding and (H) dysmenorrhea.

**Supplementary Figure 9. Forest plot.** Association between SNPs associated with coffee consumption and risk of (A) SHBG, (B) bio-T, (C) TT, (D) E_2_, (E) AMH, (F) age at menopause, (G) irregular menstrual cycle/bleeding and (H) dysmenorrhea.

**Supplementary Figure 10. Funnel plot.** Association between SNPs associated with smoking initiation and risk of (A) SHBG, (B) bio-T, (C) TT, (D) E_2_, (E) AMH, (F) age at menopause, (G) irregular menstrual cycle/bleeding and (H) dysmenorrhea.

**Supplementary Figure 11. Funnel plot.** Association between SNPs associated with alcohol drinking and risk of (A) SHBG, (B) bio-T, (C) TT, (D) E_2_, (E) AMH, (F) age at menopause, (G) irregular menstrual cycle/bleeding and (H) dysmenorrhea.

**Supplementary Figure 12. Funnel plot.** Association between SNPs associated with coffee consumption and risk of (A) SHBG, (B) bio-T, (C) TT, (D) E_2_, (E) AMH, (F) age at menopause, (G) irregular menstrual cycle/bleeding and (H) dysmenorrhea.
