# Supplementary figures and images for: The causal association between smoking initiation, alcohol and coffee consumption, and women’s reproductive health: A two-sample Mendelian randomization analysis
Source: Front Genet. 2023 Apr 6;14:1098616. doi: 10.3389/fgene.2023.1098616 (PMC10117654; doi:10.3389/fgene.2023.1098616)

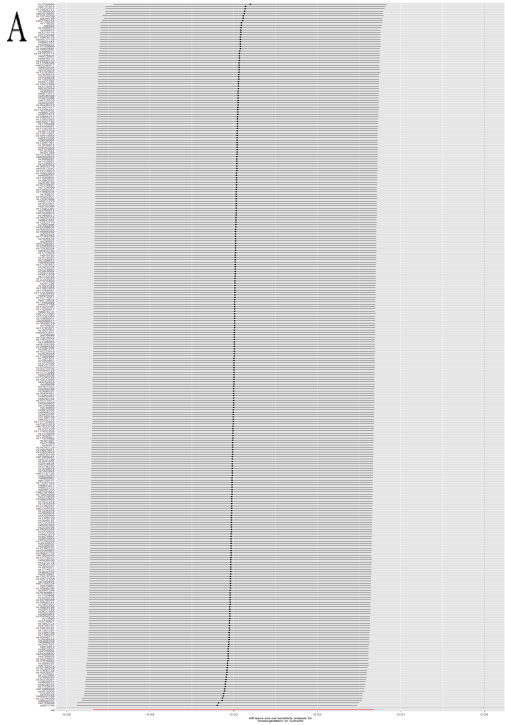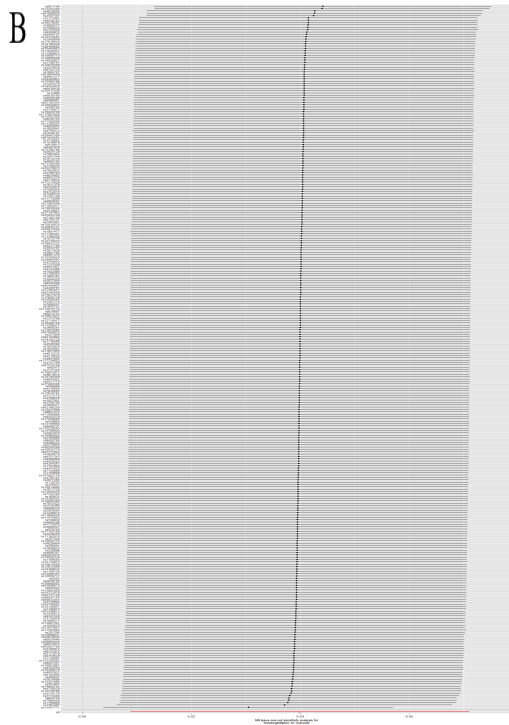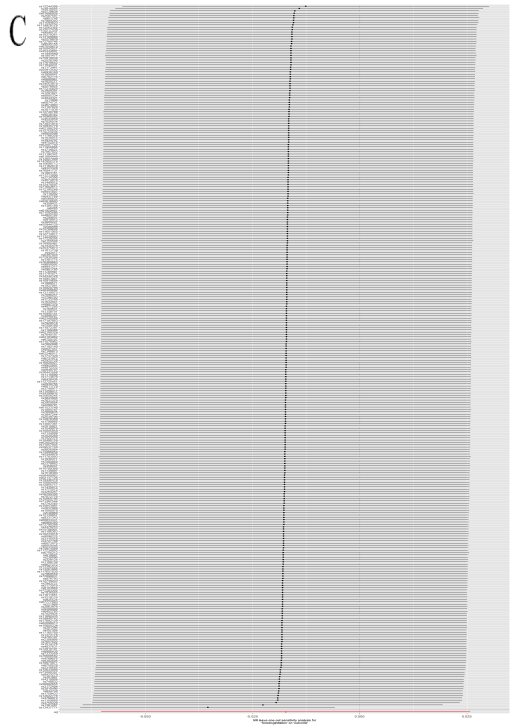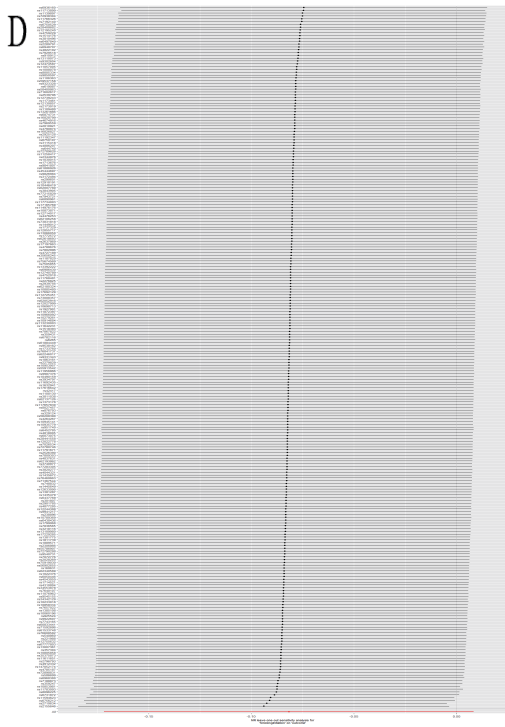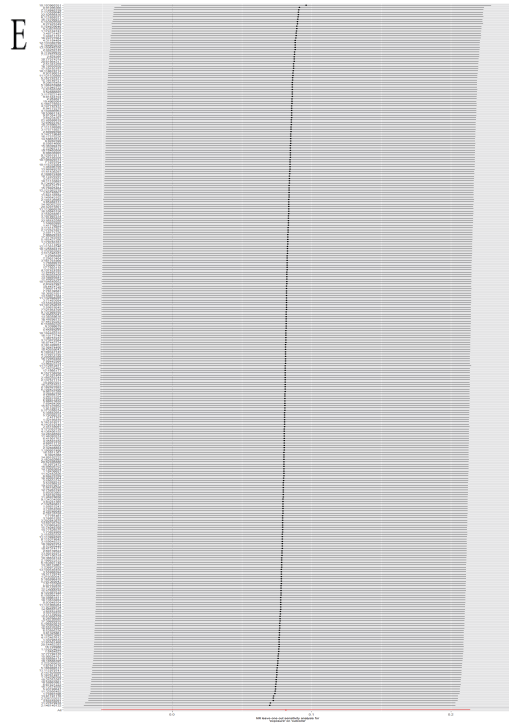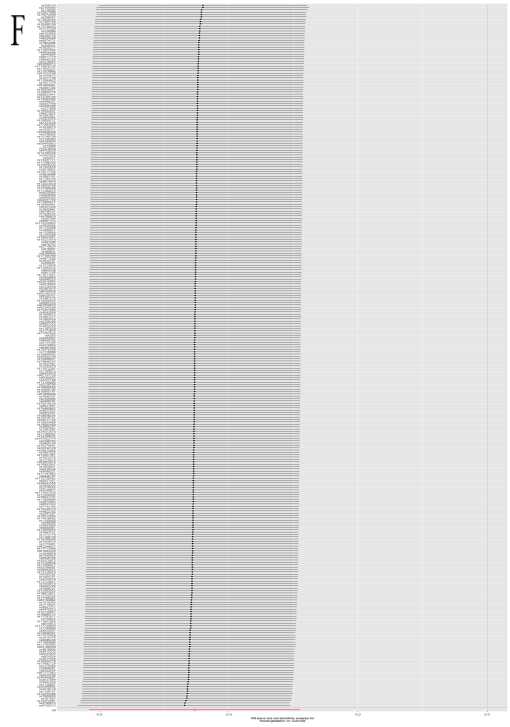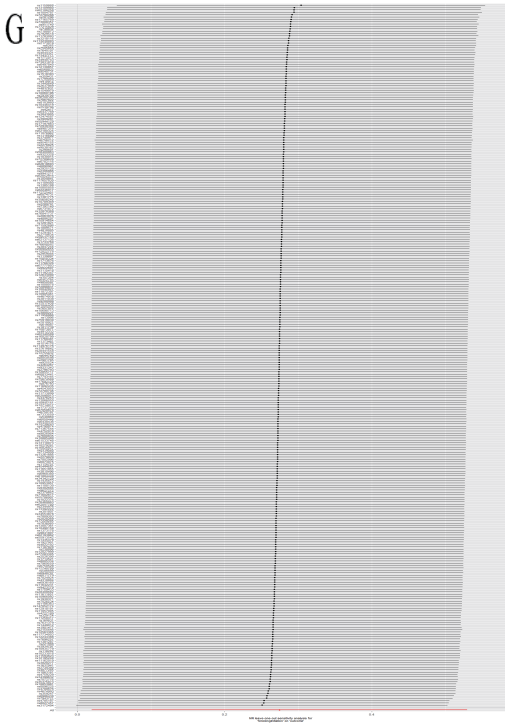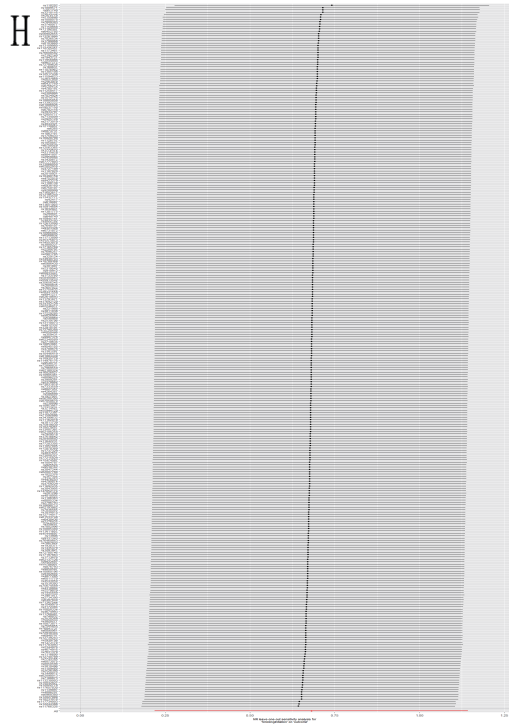

Supplement: Supplementary file 1 [file DataSheet1.ZIP › Supplementary_Materials/Supplementary Figure 1.pdf]

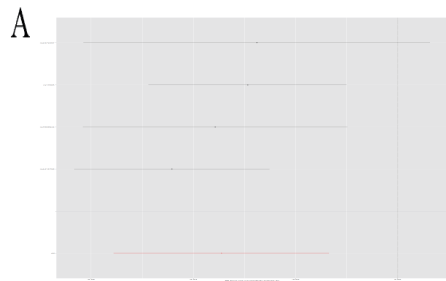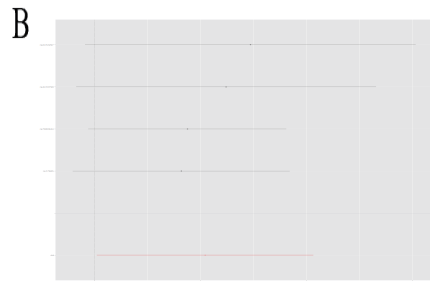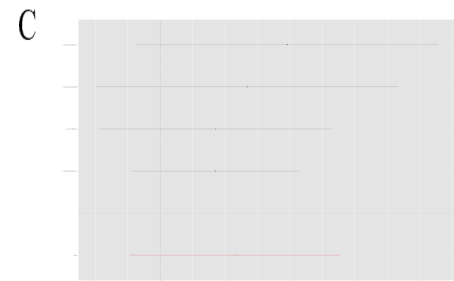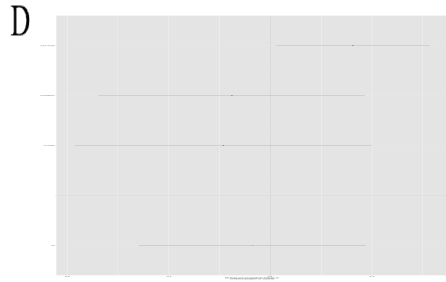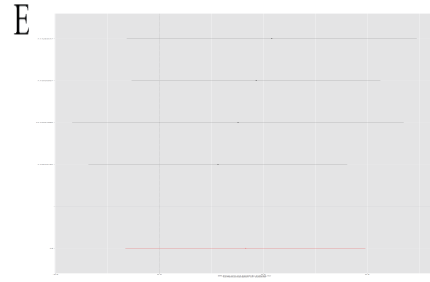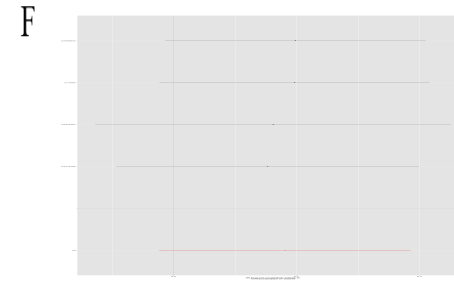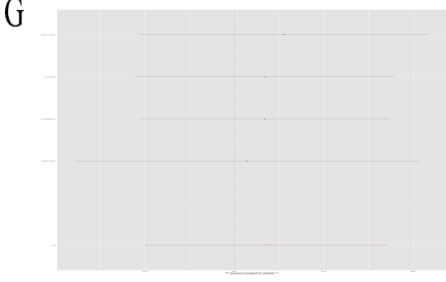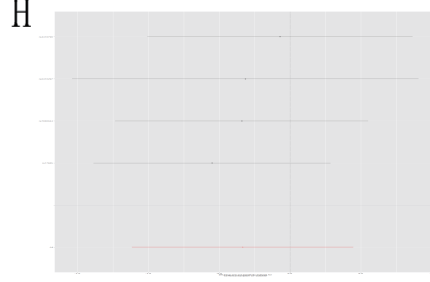

Supplement: Supplementary file 1 [file DataSheet1.ZIP › Supplementary_Materials/Supplementary Figure 3.pdf]

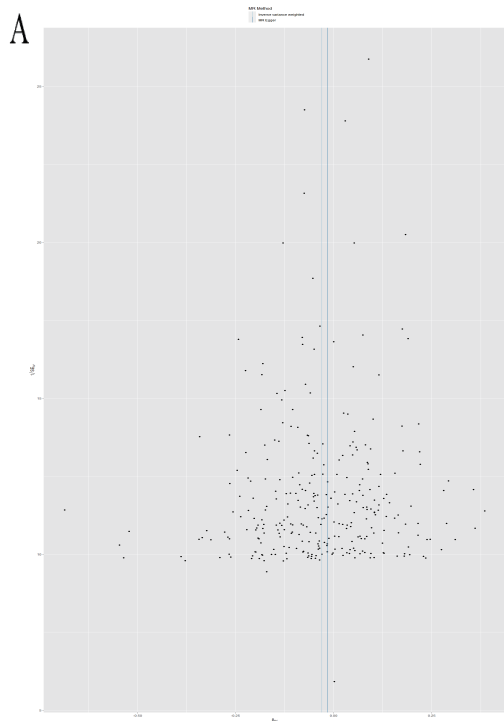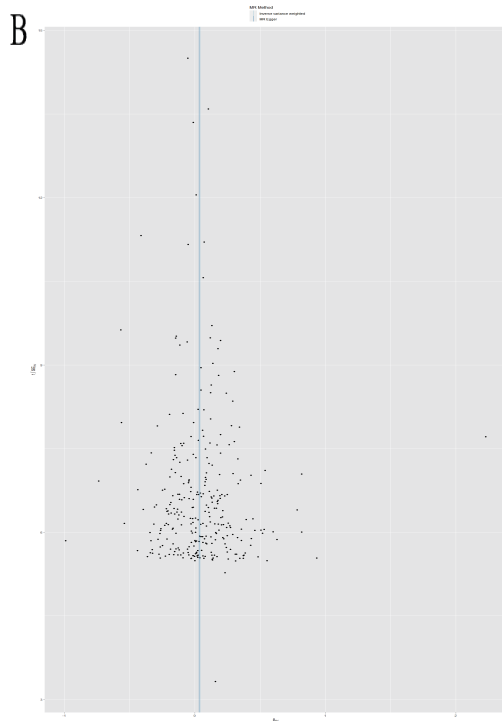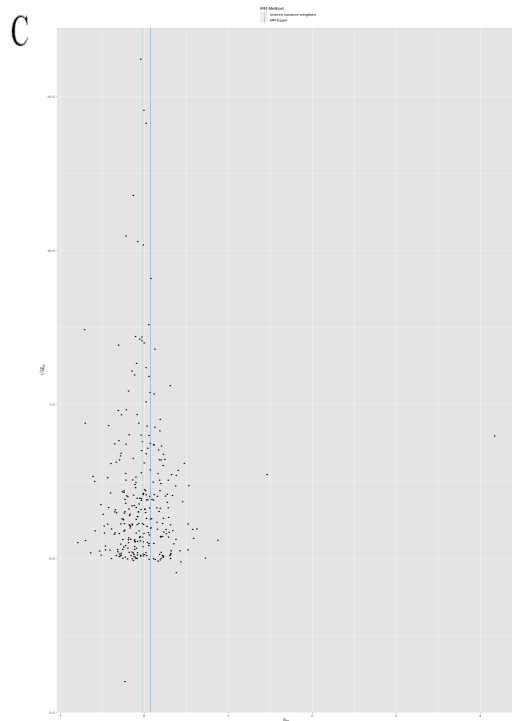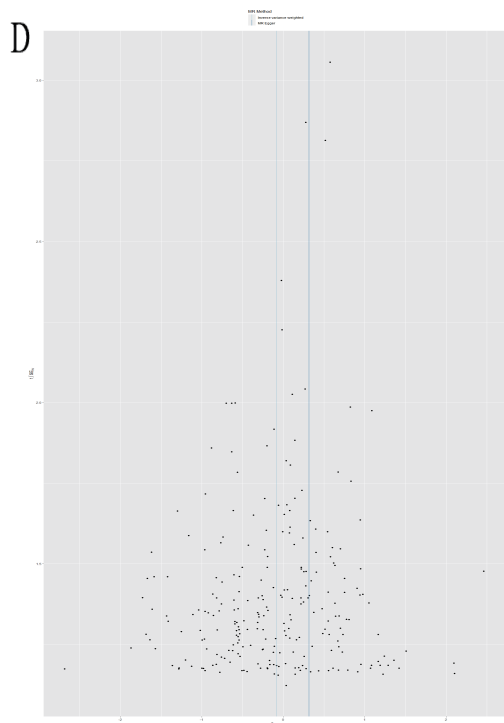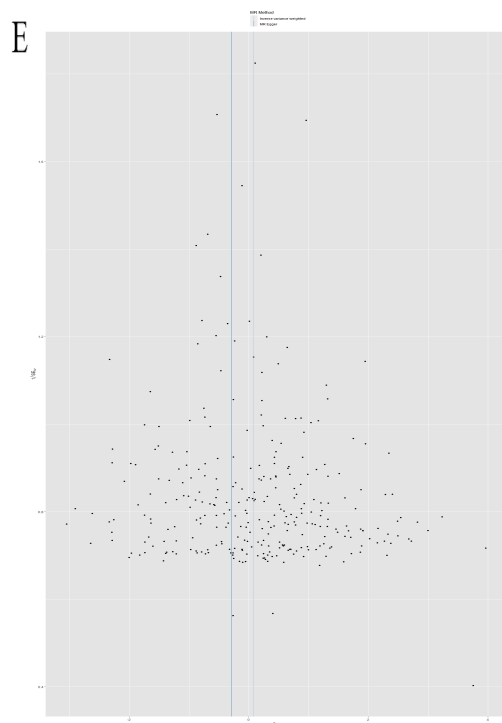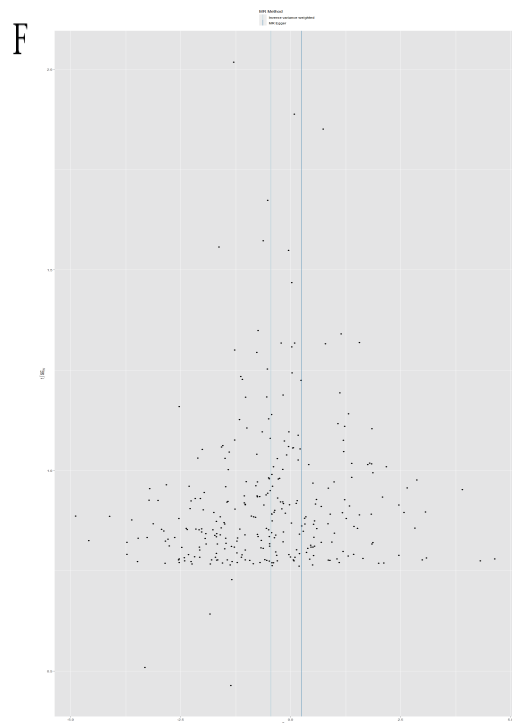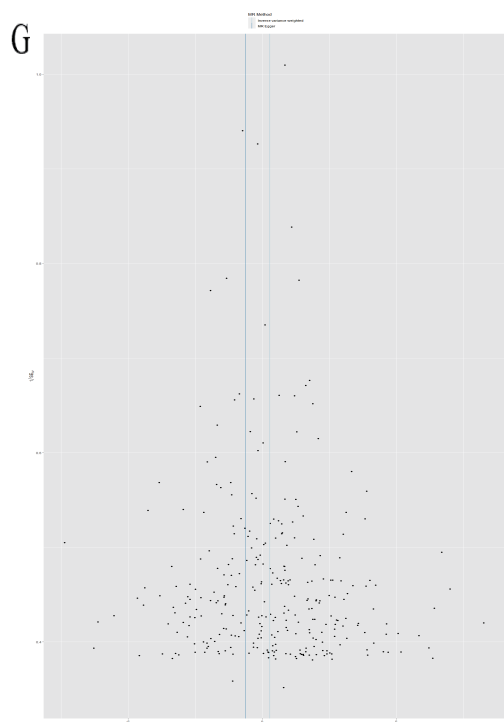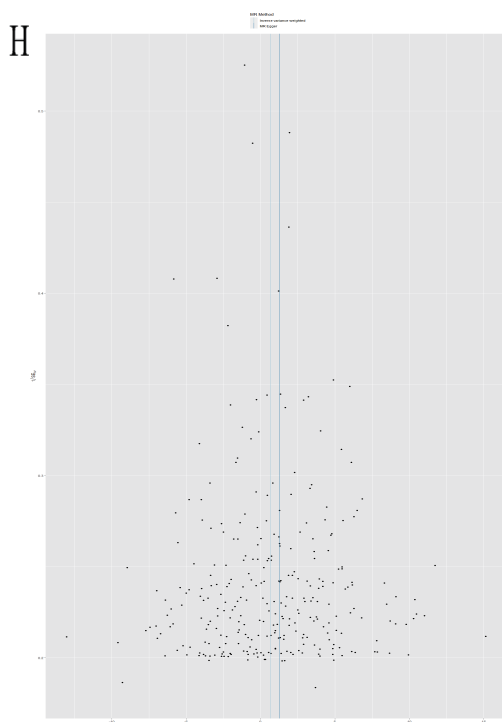

Supplement: Supplementary file 1 [file DataSheet1.ZIP › Supplementary_Materials/Supplementary Figure10.pdf]

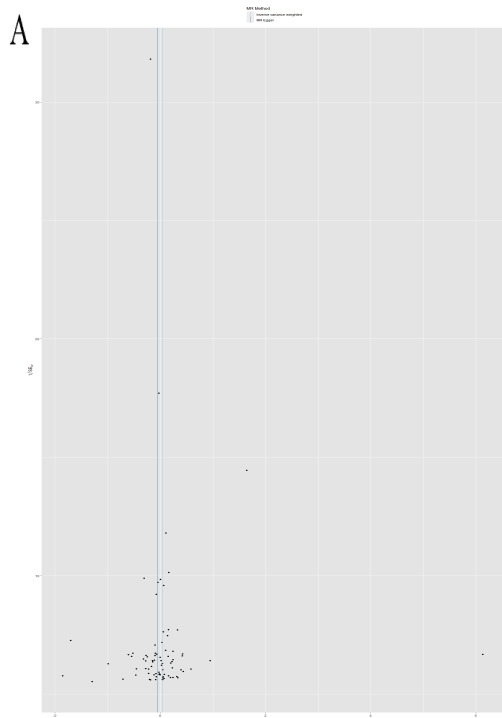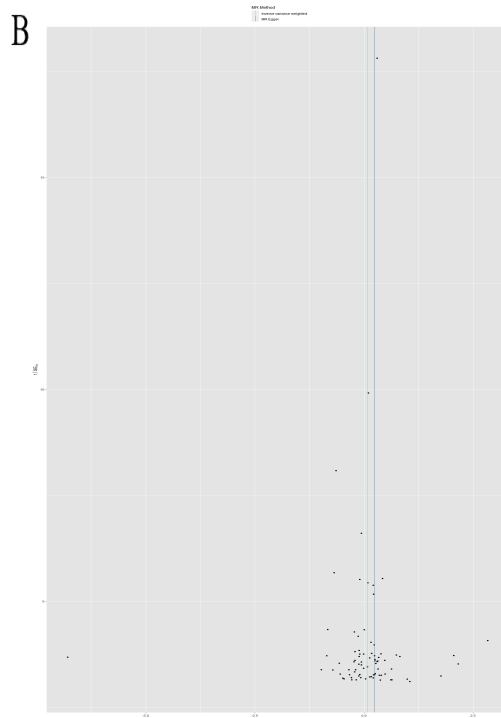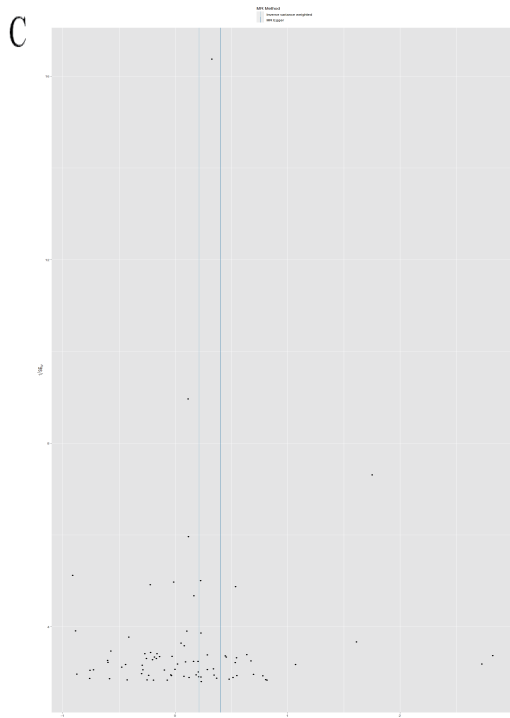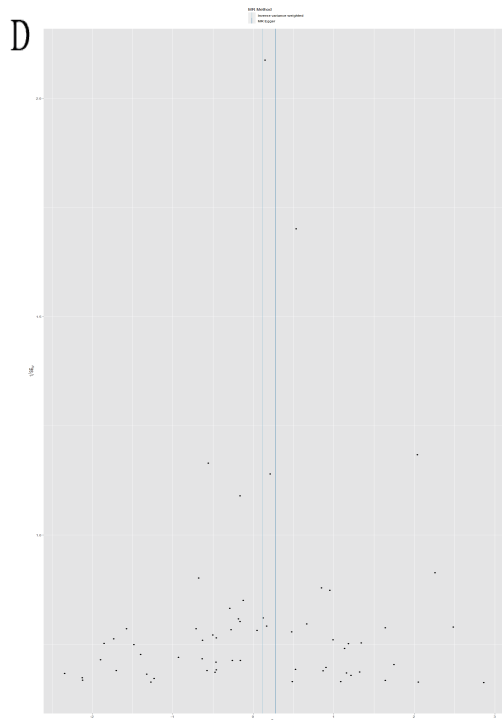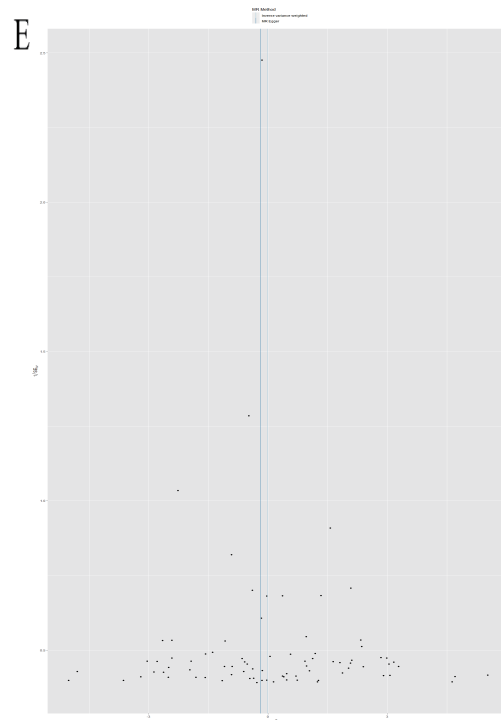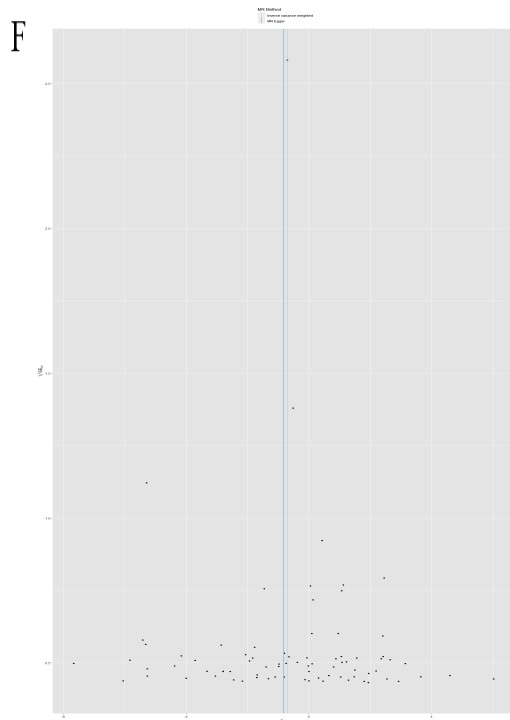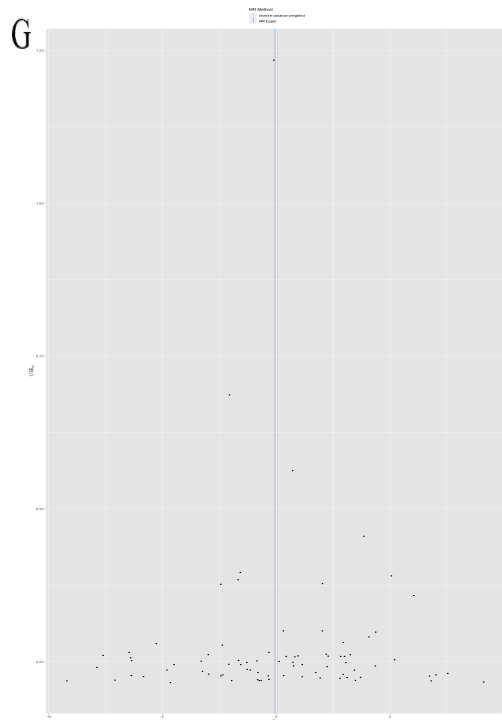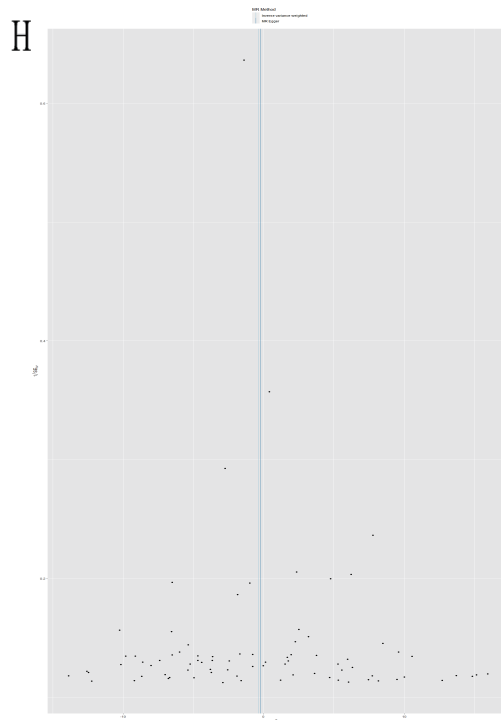

Supplement: Supplementary file 1 [file DataSheet1.ZIP › Supplementary_Materials/Supplementary Figure11.pdf]

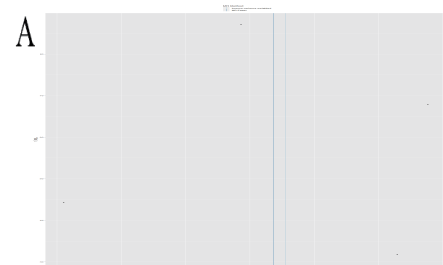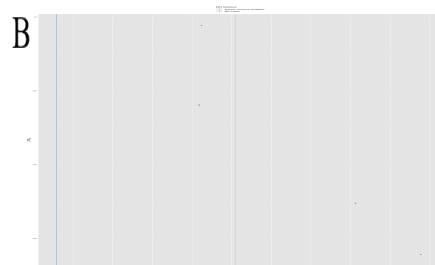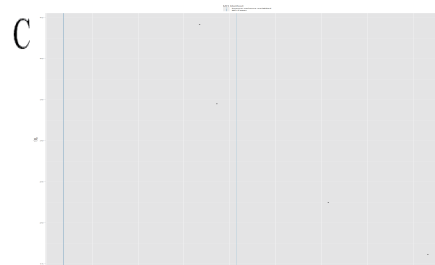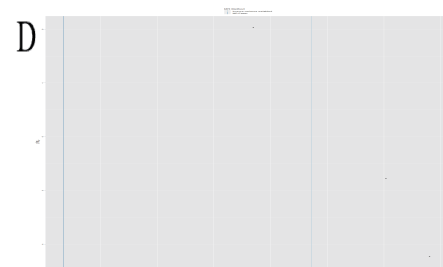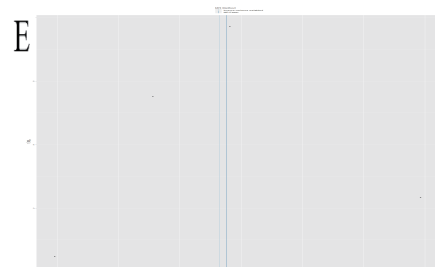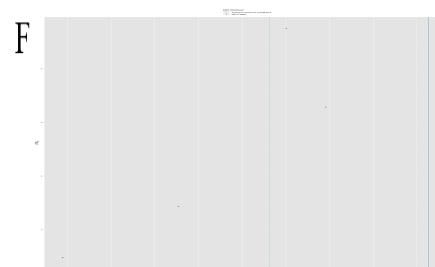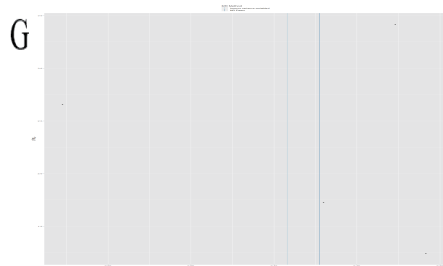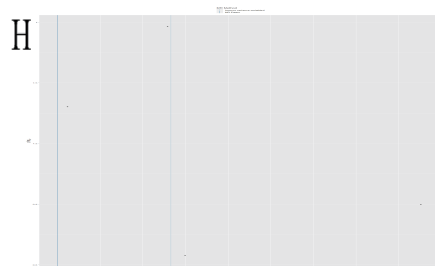

Supplement: Supplementary file 1 [file DataSheet1.ZIP › Supplementary_Materials/Supplementary Figure12.pdf]

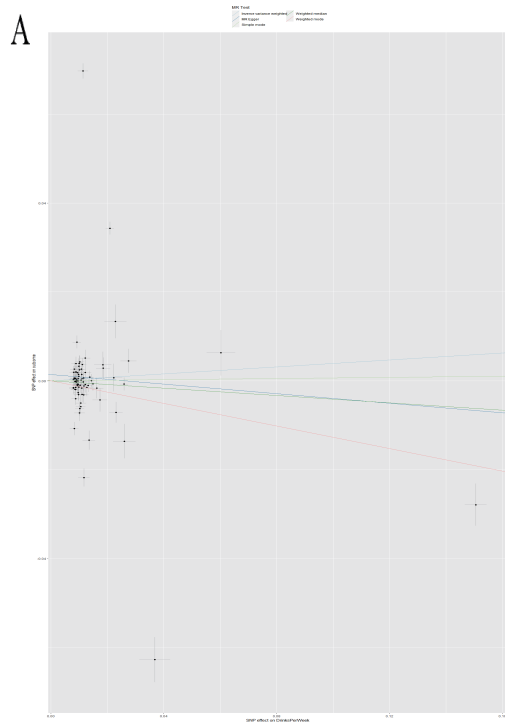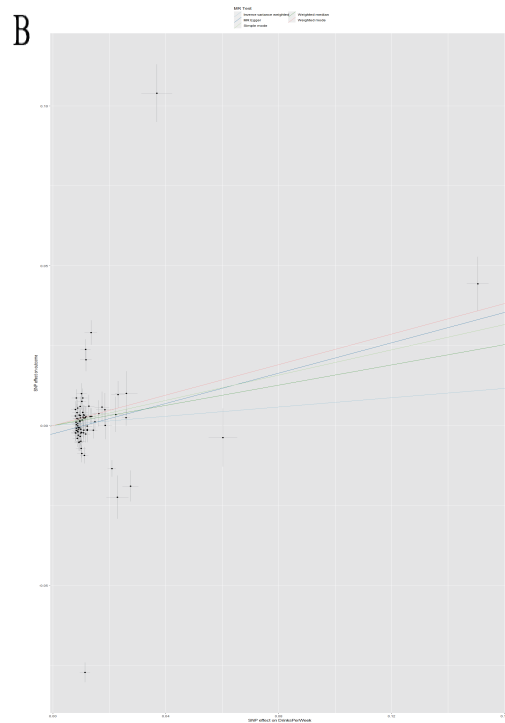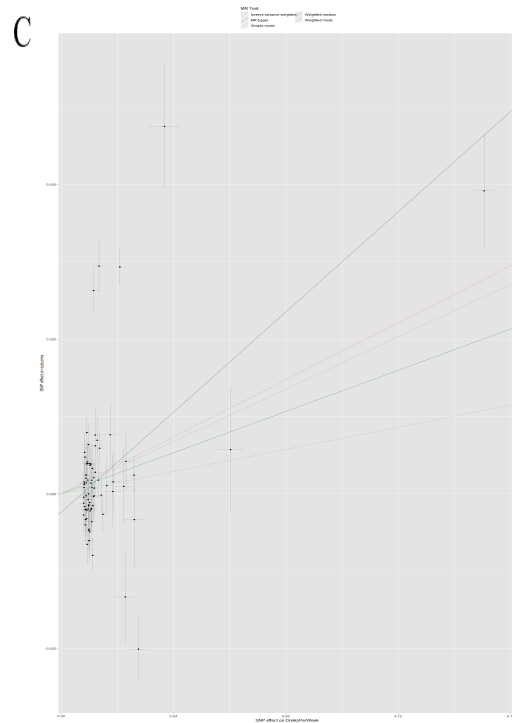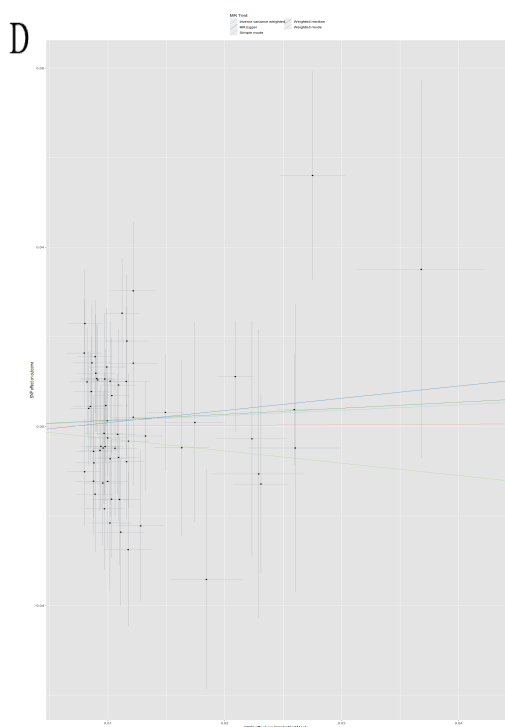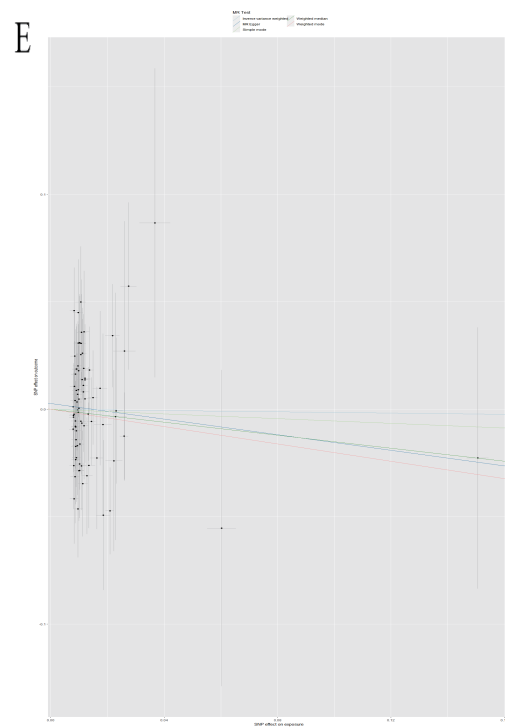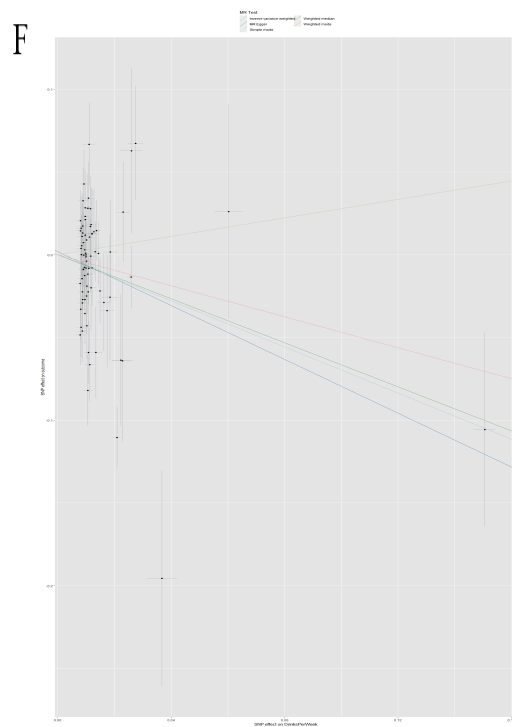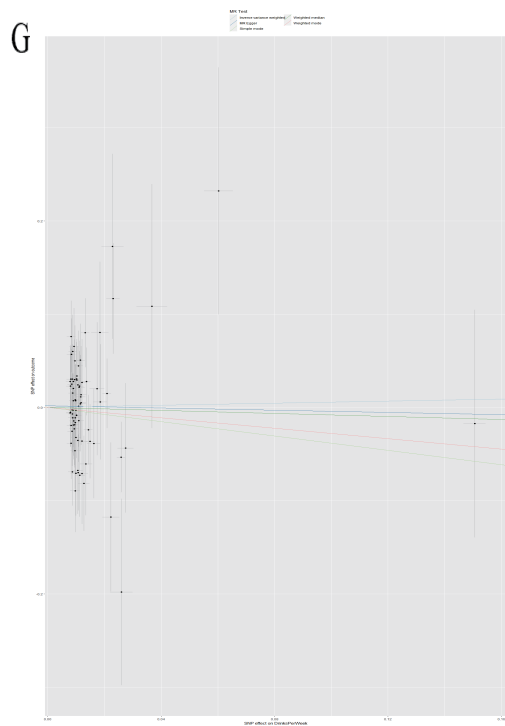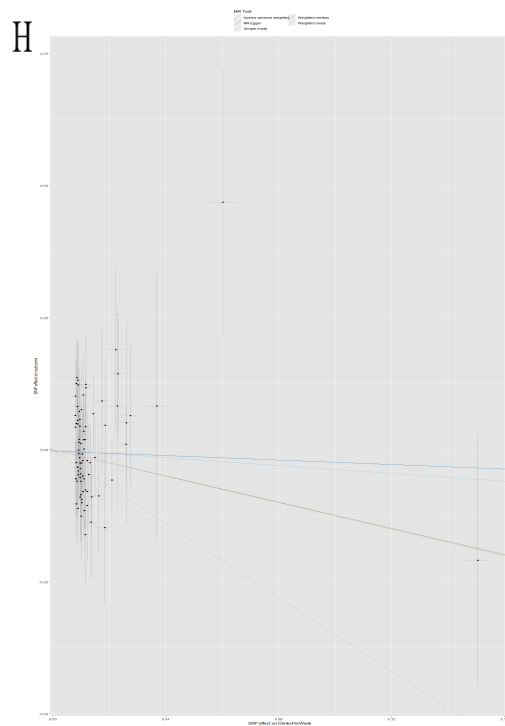

Supplement: Supplementary file 1 [file DataSheet1.ZIP › Supplementary_Materials/Supplementary Figure5.pdf]

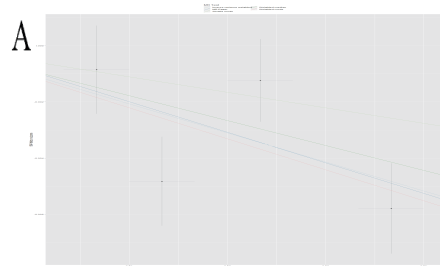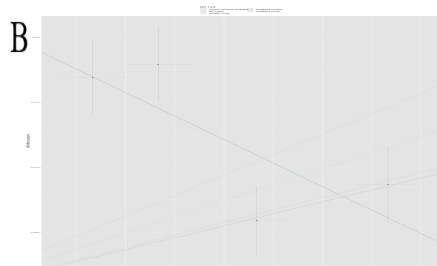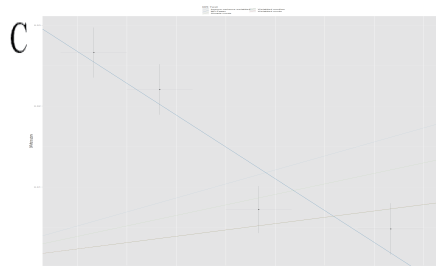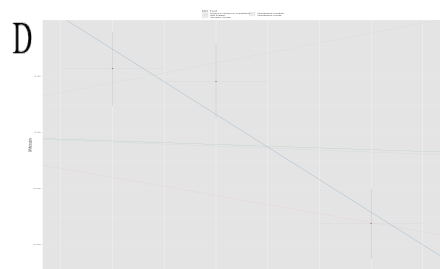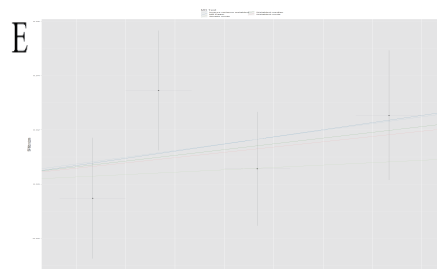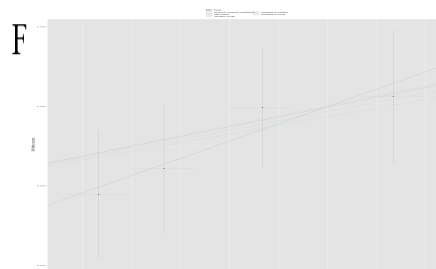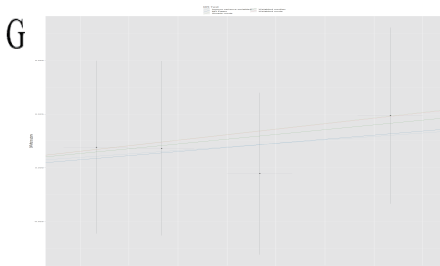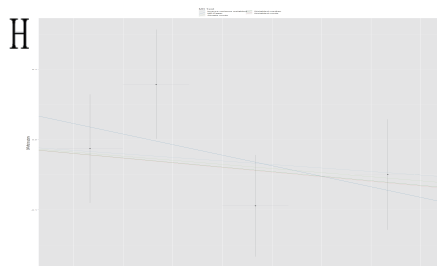

Supplement: Supplementary file 1 [file DataSheet1.ZIP › Supplementary_Materials/Supplementary Figure6.pdf]

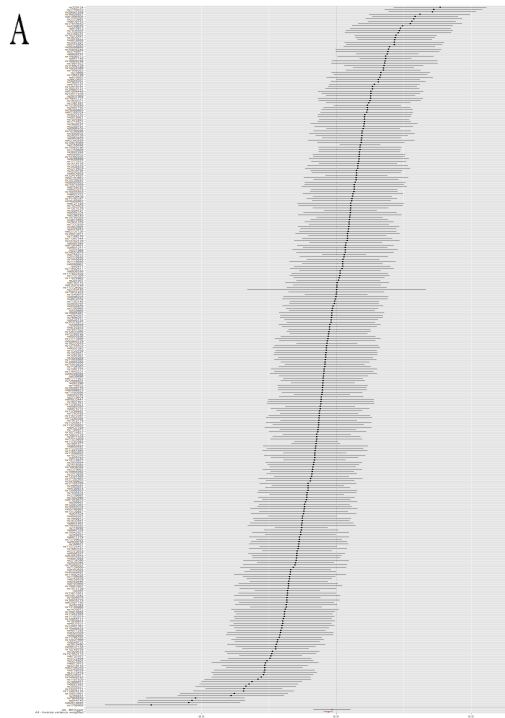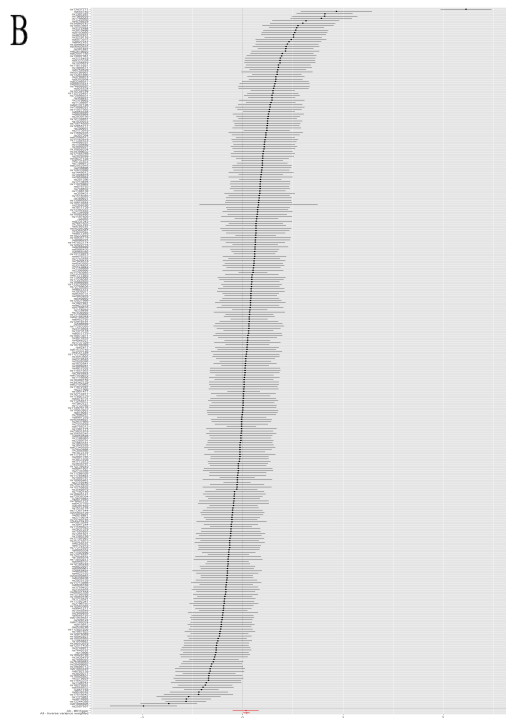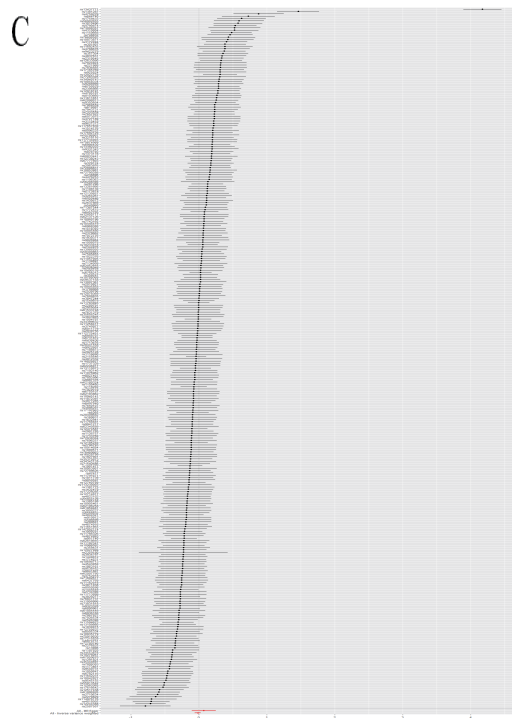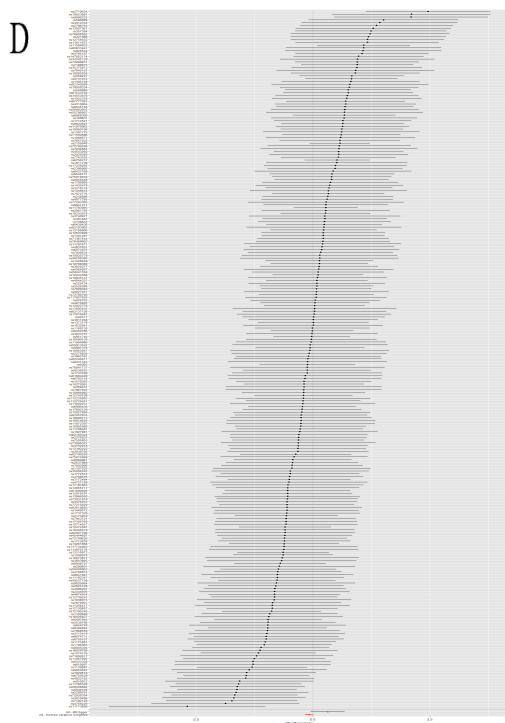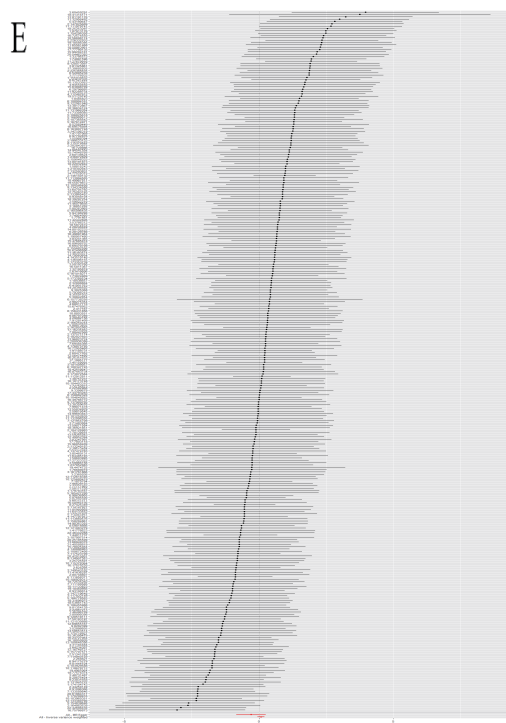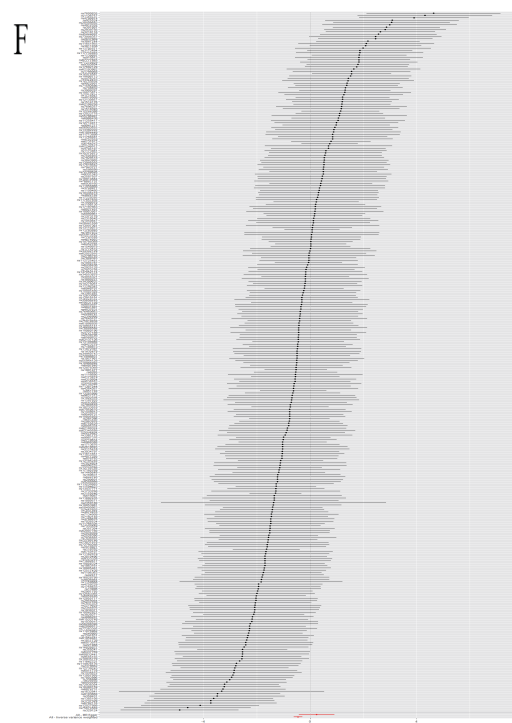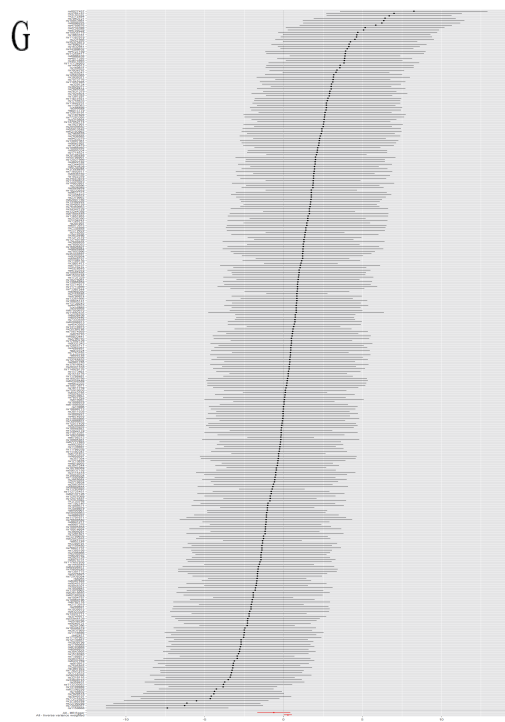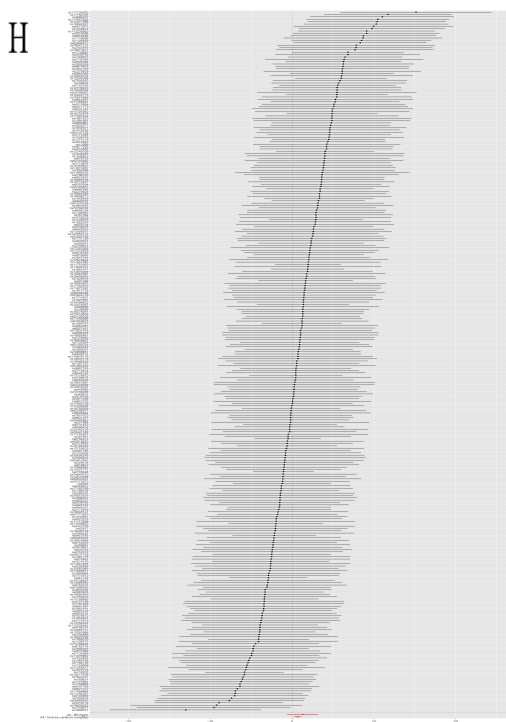

Supplement: Supplementary file 1 [file DataSheet1.ZIP › Supplementary_Materials/Supplementary Figure7.pdf]

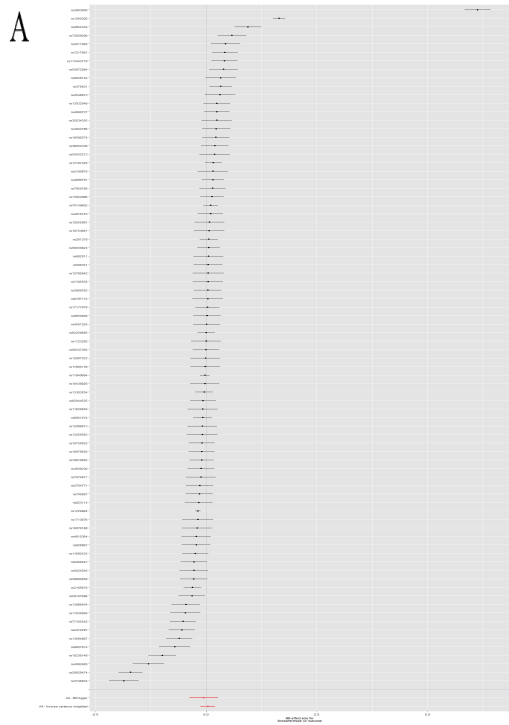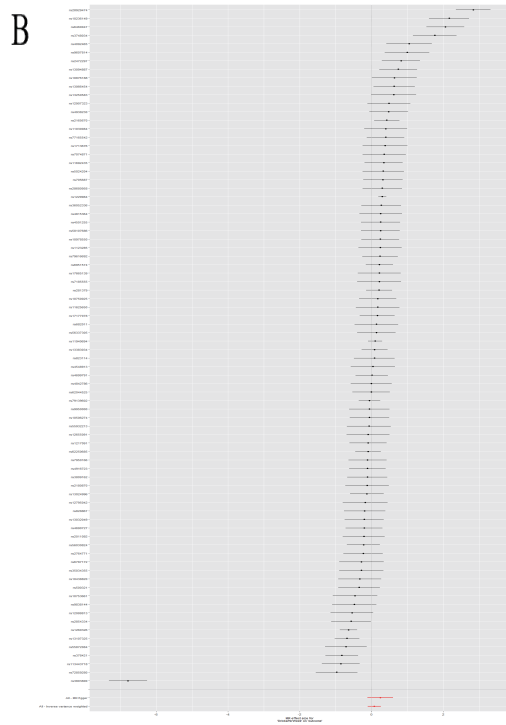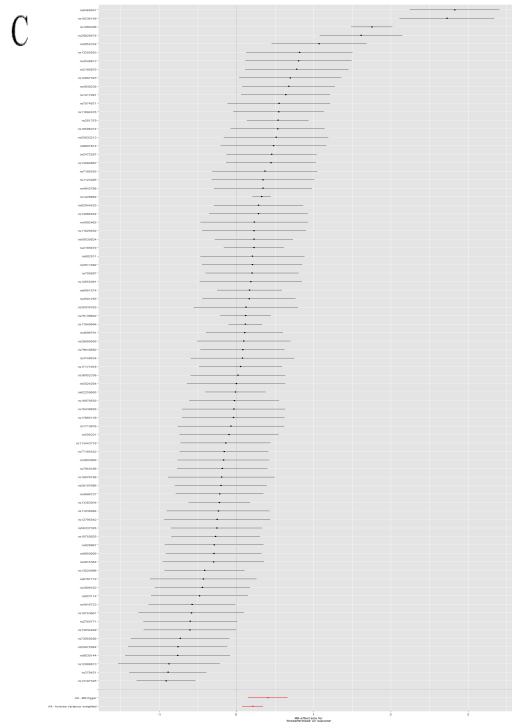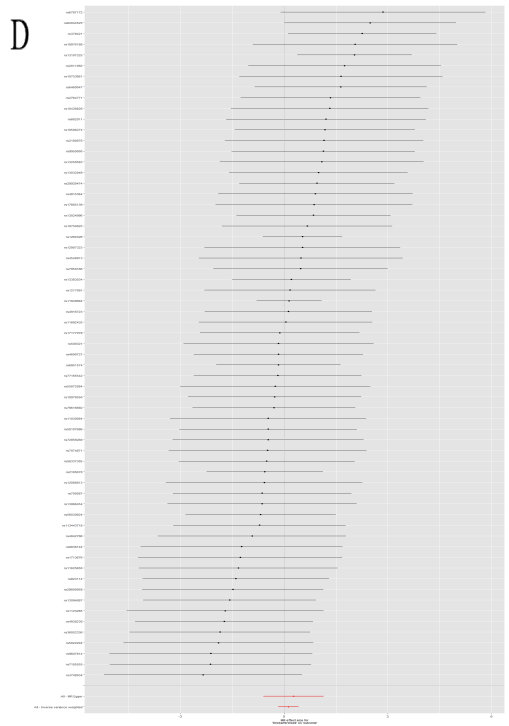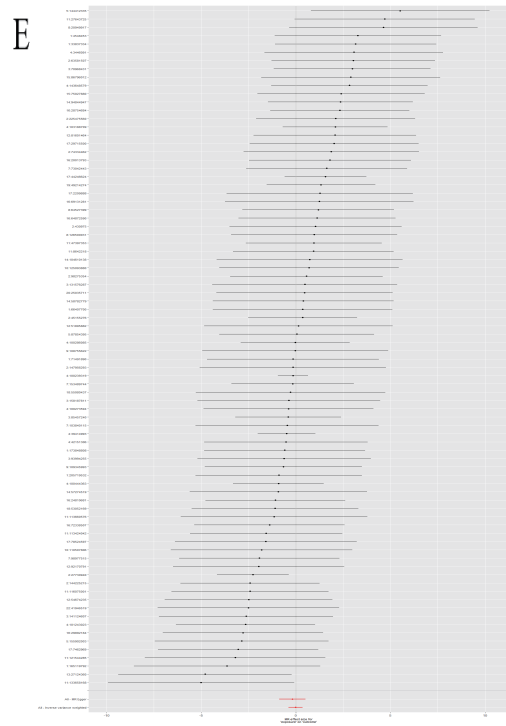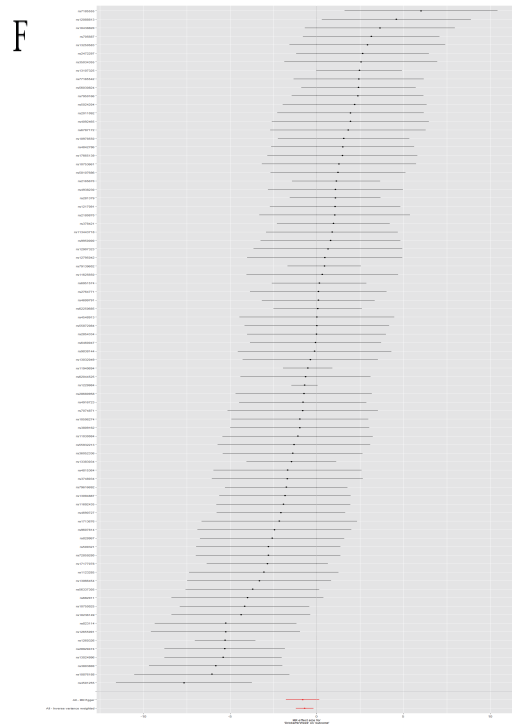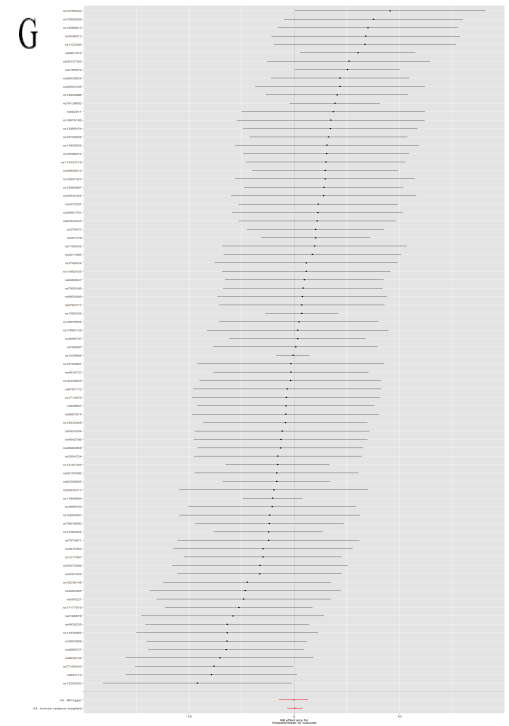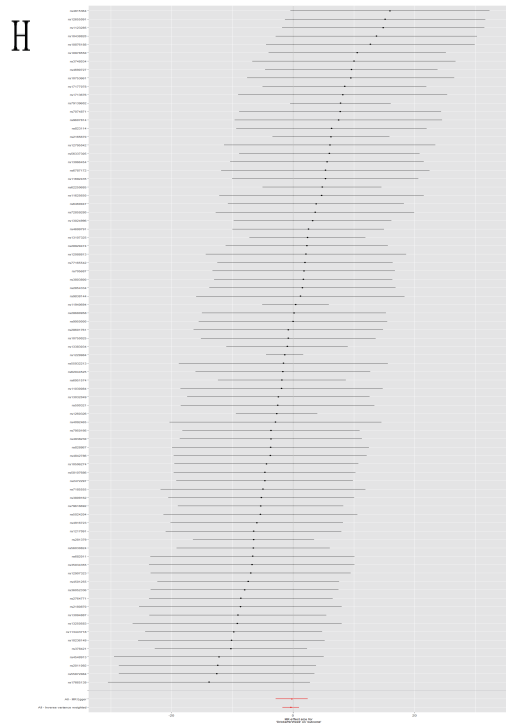

Supplement: Supplementary file 1 [file DataSheet1.ZIP › Supplementary_Materials/Supplementary Figure8.pdf]

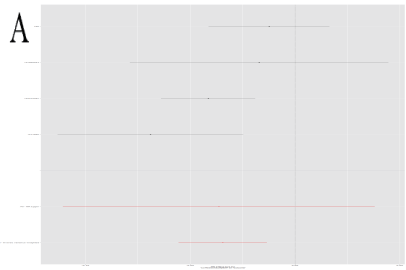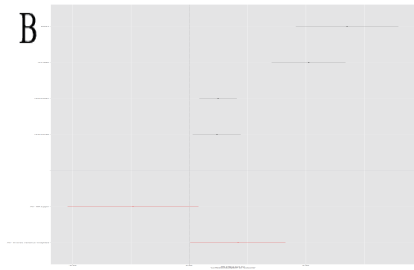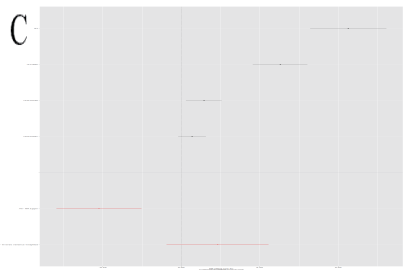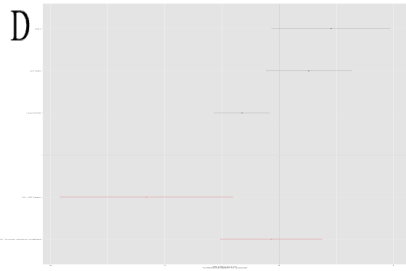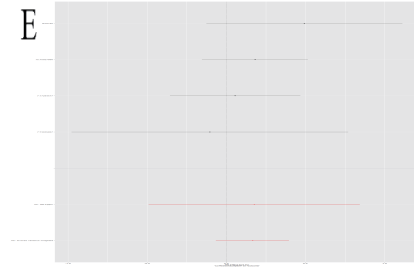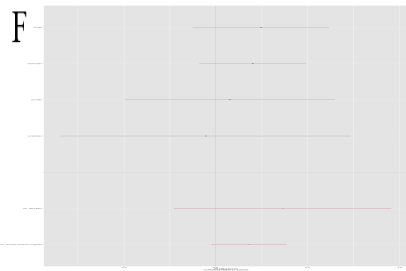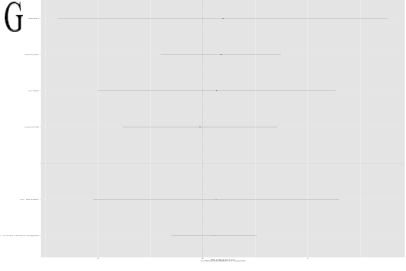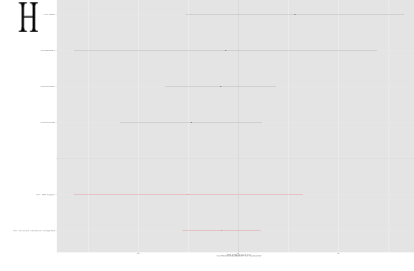

Supplement: Supplementary file 1 [file DataSheet1.ZIP › Supplementary_Materials/Supplementary Figure9.pdf]
