# Supplementary material for: The causal association between smoking initiation, alcohol and coffee consumption, and women’s reproductive health: A two-sample Mendelian randomization analysis
Source: Front Genet. 2023 Apr 6;14:1098616. doi: 10.3389/fgene.2023.1098616 (PMC10117654; doi:10.3389/fgene.2023.1098616)
Supplement: Supplementary file 1 [file Supplementary Figure 2.pdf › Supplementary_Materials/Supplementary Figure 2.pdf]

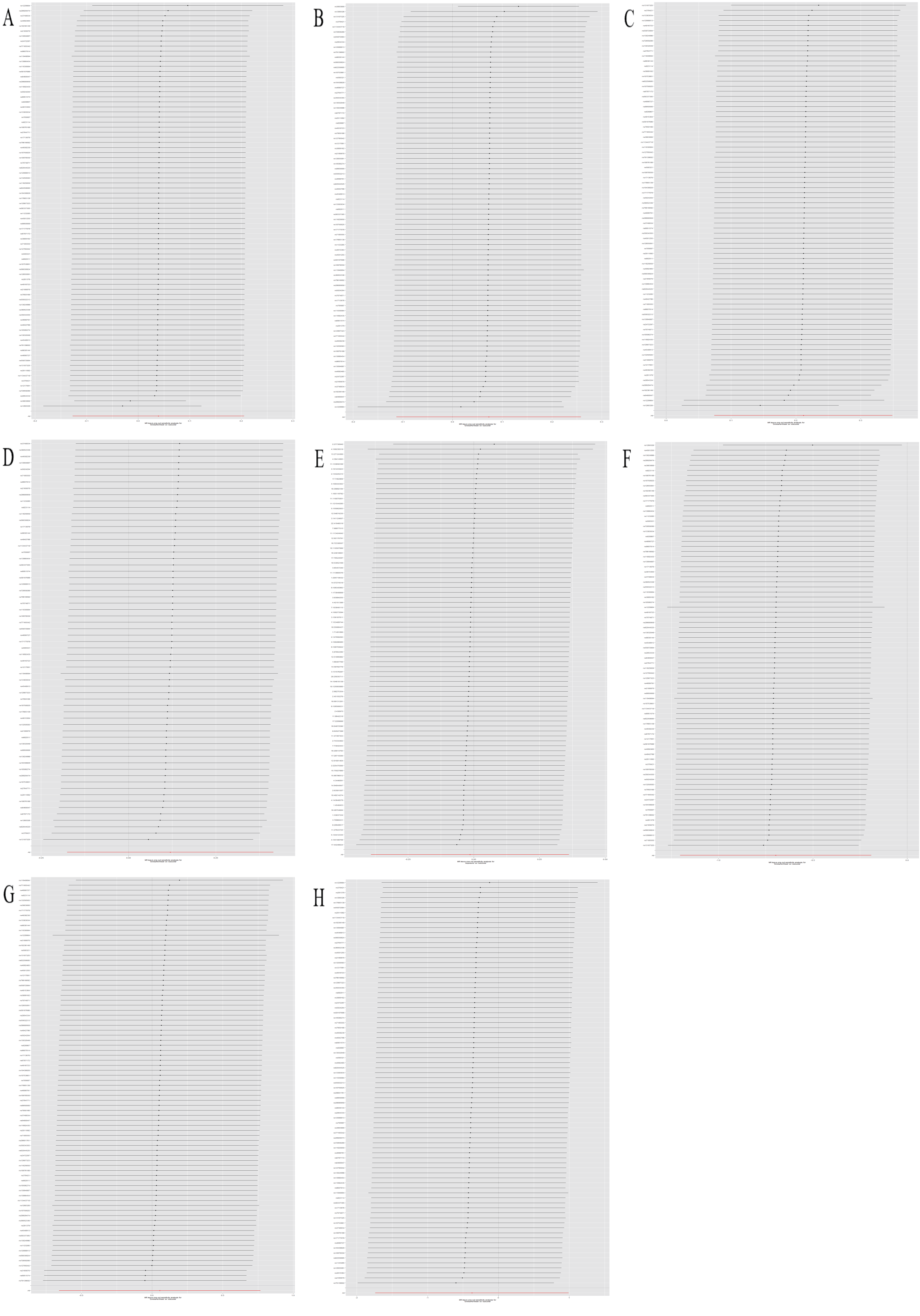
A
B
C
D
E
F
G
H
